# Wood ash biocatalyst as a novel green catalyst and its application for the synthesis of benzochromene derivatives

**DOI:** 10.1038/s41598-022-05133-x

**Published:** 2022-01-21

**Authors:** Rezvaneh Rostamian, Mohammad A. Khalilzadeh, Daryoush Zareyee

**Affiliations:** Department of Chemistry, Qaemshahr Branch, Islamic Azad University, Qaemshahr, Iran

**Keywords:** Chemistry, Materials science, Nanoscience and technology

## Abstract

Wood ash is a naturally alkaline derived substance containing organic and inorganic constituents. This study investigates the catalytic activity of wood ash as a heterogeneous catalyst for the synthesis of benzochromene derivatives. Several wood ash catalysts, comprising calcium- and potassium-rich carbonates, were prepared from different natural resources under various combustion temperatures. The prepared catalysts were characterized by Fourier transform infrared, scanning electron microscopy, energy dispersive X-ray analysis, transmission electron microscopy, and X-ray diffraction techniques. Catalytic efficiency of the resultant catalysts was tested in the synthesis of benzochromene derivatives. The experimental studies clarified that the catalyst prepared at 850 °C could efficiently expedite the formation of three-component synthesis of benzochromene derivatives in water at 80 °C with high yields. Indeed, alkali, alkaline metal, and metal oxides such as Al_2_O_3_, SiO_2_, MgO, CaO, and Fe_2_O_3_, are widely utilized as both catalyst and catalyst support in the heterogeneous catalytic processes. The prepared wood ash catalysts (possessing metal oxides, e.g., CuO, Al_2_O_3_, SiO_2_, and CaO) could effectively prompt the electrophilic activity of the carbonyl groups during the nucleophilic attack intermediate, enhancing the efficiency of the reactions.

## Introduction

Wood-ash (WA) is an organic and inorganic residue remaining from the wood or unbleached wood fiber combustion. Its physicochemical features remarkably depend on multiple parameters. In general, hardwoods can produce more ash than softwoods. Subsequently, bark and leaves produce more cash than the interior wood parts of trees^1^. Various process conditions, e.g., combustion temperature, fuel wood cleanliness, collection site, and the applied procedure significantly impact the ash production in industries. Indeed, WA composition is different according to the geographical location and the applied modification procedure^2^. WA, produced as a by-product in the wood processing industry, is produced ~ 3 million tons each year only in the United States^3^. Depending on the type of the burned wood, ~ 0.4% to 2.1% of the utilized weight is produced as ash^4^.

Several hectares of forests in different countries are burnt yearly by the wildfire producing WA as a waste. It should be noted that WA is considered as a strongly alkaline material which raises environmental issues^5^. Several studies^6,7^ indicated that calcium is the most plentiful element in WA, providing the same characteristics as the agricultural lime. In addition, ash has been considered as one of the suitable resources of potassium, phosphorus, and magnesium. Based on a study in the field^8^, diverse parameters such as various kinds of forest species, combusted plants (e.g., bark, stem, or leaves), climate, combustion conditions, etc., are effective in the composition of forest WA. Therefore, WA is broadly used in agriculture because it is a very good source of lime, potash, and other plant nutrients^9^.

The high metal content in WA indicates that it has a good potential to be used as catalytic materials for a variety of transformations. Even though several studies have been achieved on WA^8–11^, none of them have addressed the application of the WA as a heterogeneous catalyst for organic reactions, except the recently limited studies conducted on bio-diesel and esterification^11–13^.

Green methods are generally employed for various processes to decrease their expense and save resources. In addition, the use of ecological and green solvents instead of toxic solvents and applying moderate conditions and inexpensive reagents are the most important goals to develop simple and benign procedures for the synthesis of organic compounds^14^. For example, water is an inexpensive and green solvent which accelerates the rate of organic reactions for certain compounds.

Chromenes are important moieties in medicinal and organic chemistry because of their broad spectrum of biological activities including antioxidant, antimicrobial, antimalarial, anticancer, and antibacterial^15–19^. Among various chromenes, benzochromenes are highly considerable compounds because of their applicability and biological properties in variable applications^20^. The preparation of benzochromenes has been investigated using different catalysts, e.g., Zn(l-proline)_2_, 1-butyl-3-methyl imidazolium hydroxide ([bmim]OH) lipase, triethylbenzylammonium chloride (TEBA), etc.^21–25^. Although there are many novel methods to prepare these compounds, several of them have critical disadvantages such as requirement of toxic solvent, high reaction times, non-reusable catalyst, etc. Consequently, developing efficient and inexpensive catalysts presenting high catalytic activity for the preparation of benzochromenes is highly desirable^14^. Indeed, the preparation of benzochromenes by multicomponent reactions (MCR) has garnered much attention because of good product yield and their applicability.

In continuation of our studies to explore new preparation procedure for main organic compounds^26–32^, we introduce a green method for the synthesis of benzochromene derivatives by an efficient three component reaction of 1-(6-hydroxy-2-isopropenyl-1-benzofuran-yl)-1-ethanone or euparin **1**^33^, aldehydes **2**, alkyl bromides **3** and triphenylphosphine **4** in the presence of water extract WA (WEWA) as a catalyst in water at 70 °C with good yields. In addition, the antioxidant activities of some of the synthesized derivatives were studied by ferric ion reducing power test and DPPH radical scavenging. To the best of our knowledge, the application of WA as a catalyst for MCR reactions has not previously been reported. The aim of this study is to acquire an active and inexpensive catalyst from waste WA for the synthesis of some benzochromene derivatives. For this purpose, several catalysts were prepared from different WA and characterized by the latest analytical techniques.

## Results and discussion

### Characterization of WA

#### Basicity

Our initial studies focused on soluble basicity measurement with the aim of finding optimum conditions. It was found that the source of the wood and combustion temperature both have a deep influence on basicity. Therefore, all the WA which prepared at four different burning temperatures are provided for pH measurement and the results of the WA samples are tabulated in Table 1.

**Table 1:** Soluble alkalinity of WA catalysts.

Sample	WA	Burning temp. (°C)	Obtaining ash (%)	pH
PiA_450_	Pine	450	12.4	9.45 ± 0.08
PiA_650_	Pine	650	9.1	10.04 ± 0.13
PiA_850_	Pine	850	5.7	11.65 ± 0.09
PiA_1050_	Pine	1050	3.9	10.94 ± 0.08
ROA_450_	R. olive	450	14.3	11.67 ± 0.09
ROA_650_	R. olive	650	10.4	12.33 ± 0.16
ROA_850_	R. olive	850	6.4	12.79 ± 0.04
ROA_1050_	R. olive	1050	4.7	12.23 ± 0.12
PoA_450_	Poplar	450	12.2	9.32 ± 0.11
PoA_650_	Poplar	650	9.3	10.12 ± 0.06
PoA_850_	Poplar	850	5.2	11.43 ± 0.09
PoA_1050_	Poplar	1050	3.8	10.76 ± 0.02

It is observed that both source of wood and combustion temperature affect pH or basicity of the ash samples. The results indicated that the pH of Russian olive ash (ROA) is much more than pine ash (PiA) and poplar ash (PoA) and the pH of the ash samples increases with increasing of burning temperature for example for PiA from 9.45 to 11.65 from 450 to 850 °C and decreases to 10.94 with increase of combustion temperature (above 850 °C). The pH of the ROA_850_ (Russian olive Ash at 850 °C) has the highest value 12.79.

**Figure 1:**
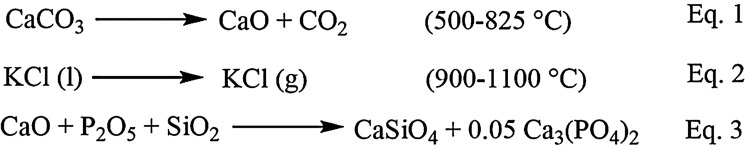
Thermal decomposition of CaCO_3_ and formation of CaSiO_4_.

Thermal decomposition of CaCO_3 _(825 °C) to CaO is perhaps the possible reason for higher basicity with the increase of temperature, which because of higher solubility of CaO in water than CaCO_3_, an increase in basicity value is resulted^34^. But the reason that at temperatures above 1000 °C, the pH shows a great decrease is because of the formation of a highly stable silicate phase via the interaction of metal oxides (e.g., SiO_2_ and CaO). The reason for the decrease in basicity is that the stable silicates are less soluble in the water (Fig. 1)^35^.

#### SEM–EDX analysis

**Figure 2:**
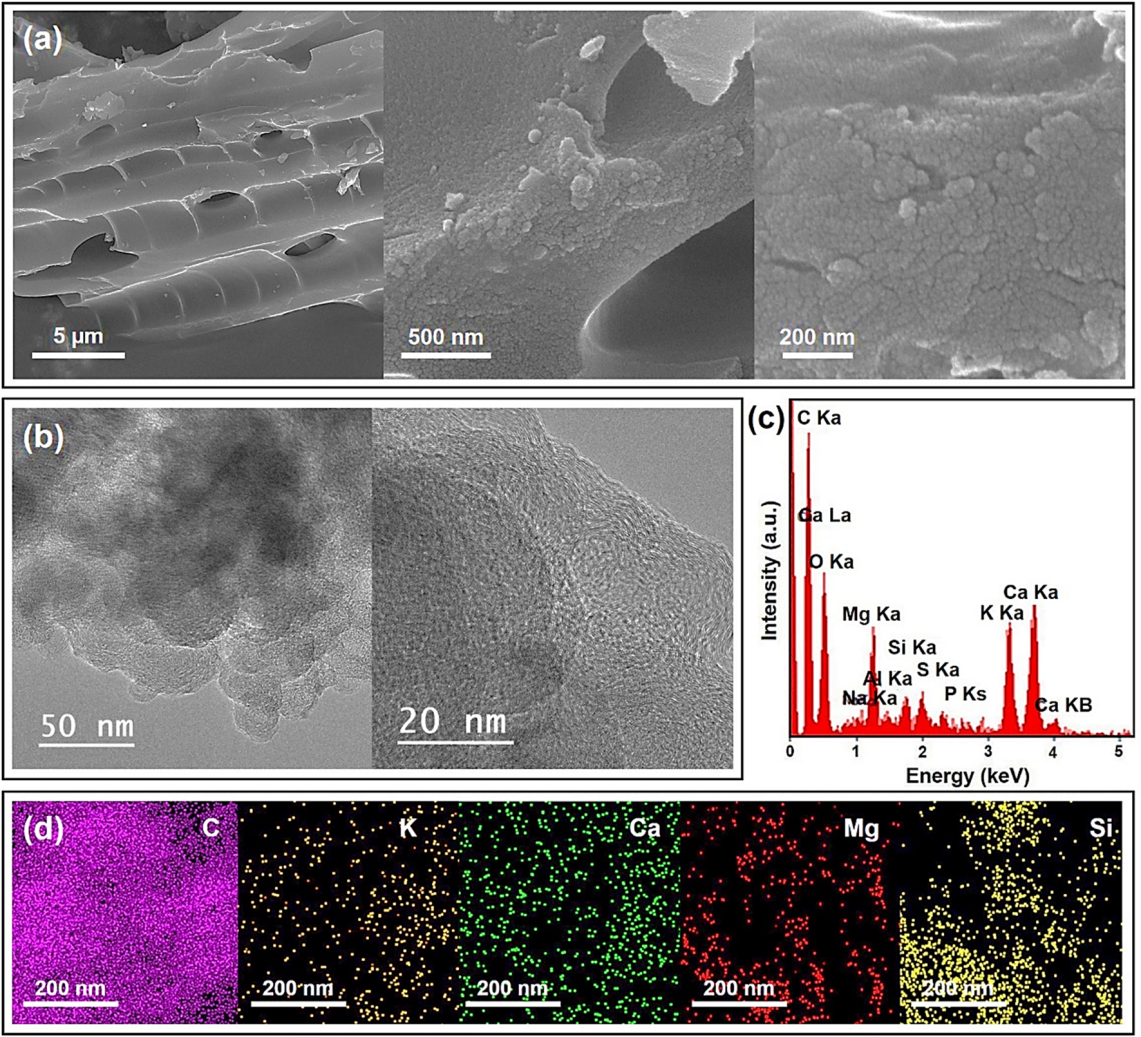
(**a**) FE-SEM, (**b**) TEM and HRTEM images, (**c**) EDX analysis and (**d**) EDX elemental mapping of WA prepared at 850 °C.

Figure 2a shows the scanning electron microscopy (SEM) analysis of WA from the combustion of the Russian olive wood at 850 °C (ROA_850_). The SEM image of the ROA_850_ illustrates the porous and spongy nature with rough surfaces and high surface areas^12^ in WA particles. Most of the particles characterize sphere-shaped structure. Transmission electron microscopy (TEM) and high-resolution TEM (HRTEM) images show high crystallinity of the prepared wood ash at 850 °C (Fig. 2b). The energy dispersive X-ray (EDX) analysis used to provide the elemental composition and to evaluate a structural vision of the ROA_850_ sample as shown in Fig. 2c. The elements identified were potassium (K), calcium (Ca), magnesium (Mg), phosphorus (P), oxygen (O), carbon (C), sulfur (S), aluminum (Al), silicon (Si), and sodium (Na). As expected, the main elements of C, and O were consistently dispersed on the surface of the RO WA. Additionally, according to the EDX analysis and elemental mapping, the main elemental compositions of ROA_850_ were mainly C, K, Ca, Mg, and Si, which indicates the presence of calcium and potassium rich carbonates and oxides on the surface of RO WA (Fig. 2d).

#### X-ray diffraction

**Figure 3:**
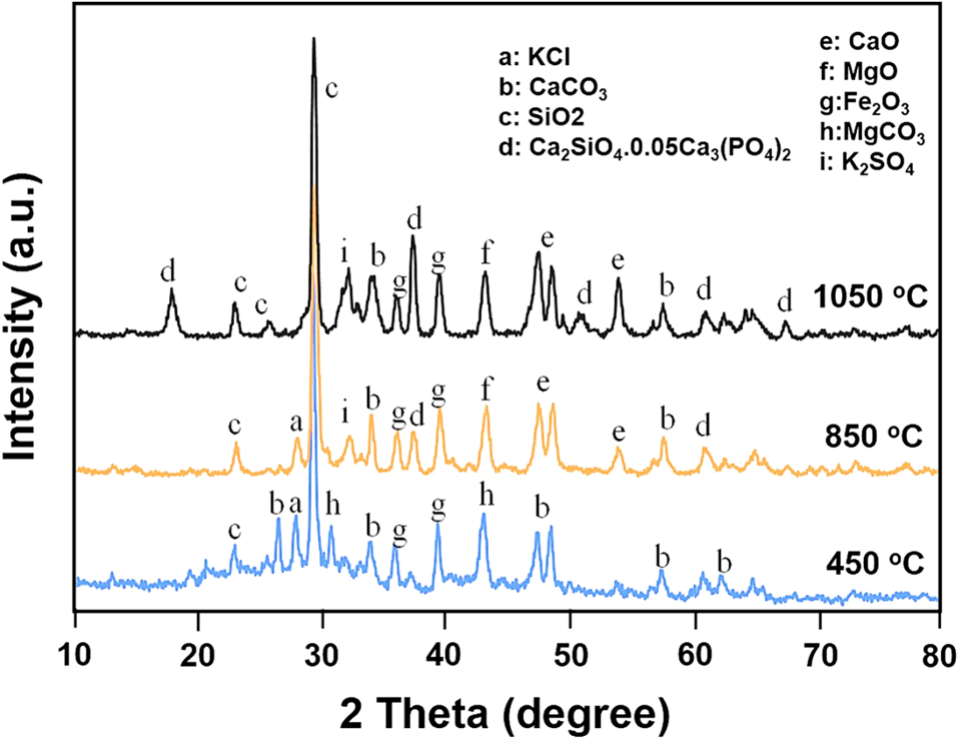
XRD pattern of WA at different combustion temperatures.

The X-ray diffraction (XRD) patterns of the Russian olive WA prepared at different combustion temperatures (ROA_450_, ROA_850_, and ROA_1050_) are shown in Fig. 3. The XRD pattern of ROA_450_ shows the presence of CaCO_3_, SiO_2_, Fe_2_O_3_, KCl, and MgCO_3_ components. On burning of Russian olive wood at 850 °C (ROA_850_), the corresponding XRD pattern indicates the presence of KCl, CaO, Fe_2_O_3_, MgO, CaCO_3_, K_2_SO_4_, and Ca_2_SiO_4_.0.05Ca_3_(PO_4_)_2_ compounds. After combustion at higher temperature at 1050 °C (ROA_1050_), XRD pattern indicates that number and intensity of peaks related to Ca_2_SiO_4_.0.05Ca_3_(PO_4_)_2_ compound increases and is found as the main component.

#### X-ray fluorescence

It is not clear by what mechanism mineral compounds are formed as ash through burning of wood although it is acceptable to believe that the exchange depends upon the burning temperature and current atmosphere. Table 2 shows chemical composition analysis of Russian olive WA prepared at different combustion temperatures (ROA_450_, ROA_850_, and ROA_1050_). The minerals in WA determined by X-ray fluorescence (XRF) found to be Na_2_O, K_2_O, MgO, BaO, CaO, SiO_2_, Al_2_O_3_, P_2_O_5_, Fe_2_O_3_, and MnO_2_. As shown in Table 2, in case of combustion Russian olive WA samples, the changes under the combustion temperature from 450 to 1050 °C causes the modification in composition of the prepared ashes. At 850 °C combustion temperature, the increase in CaO content was observed mainly because of decay of CaCO_3_ (Eq. 1), also verified by the XRD pattern of ROA_850_ (Fig. 3). At higher temperature, the percentages of Na_2_O and MgO decrease due to their carbonates decomposition to oxides and the subsequent volatilization^36^. The decrease in potassium percentage is predominantly because of vaporization of KCl (Eq. 2). The probable cause for rising CaO content may be because of non-volatility of CaO from the WA^36^.

**Table 2:** Chemical composition of the WA from Russian olive in different combustion temperatures.

Sample	Na_2_O	K_2_O	MgO	BaO	CaO	SrO	CuO	SiO_2_	Al_2_O_3_	P_2_O_5_	Fe_2_O_3_	MnO_2_	TiO_2_	SO_3_
ROA_450_	0.7	20.6	7.4	1.7	45.6	1.6	–	7.2	1.6	4.3	3.6	0.3	0.2	5.2
ROA_850_	0.5	14.5	9.64	0.2	61.5	2.1	0.1	2.9	0.51	2.5	0.9	0.3	0.1	4.3
ROA_1050_	0.2	4.2	3.4	1.6	64.9	3.5	1.2	3.5	3.2	2.3	4.2	1.6	0.5	5.7

#### FT-IR analysis

**Figure 4:**
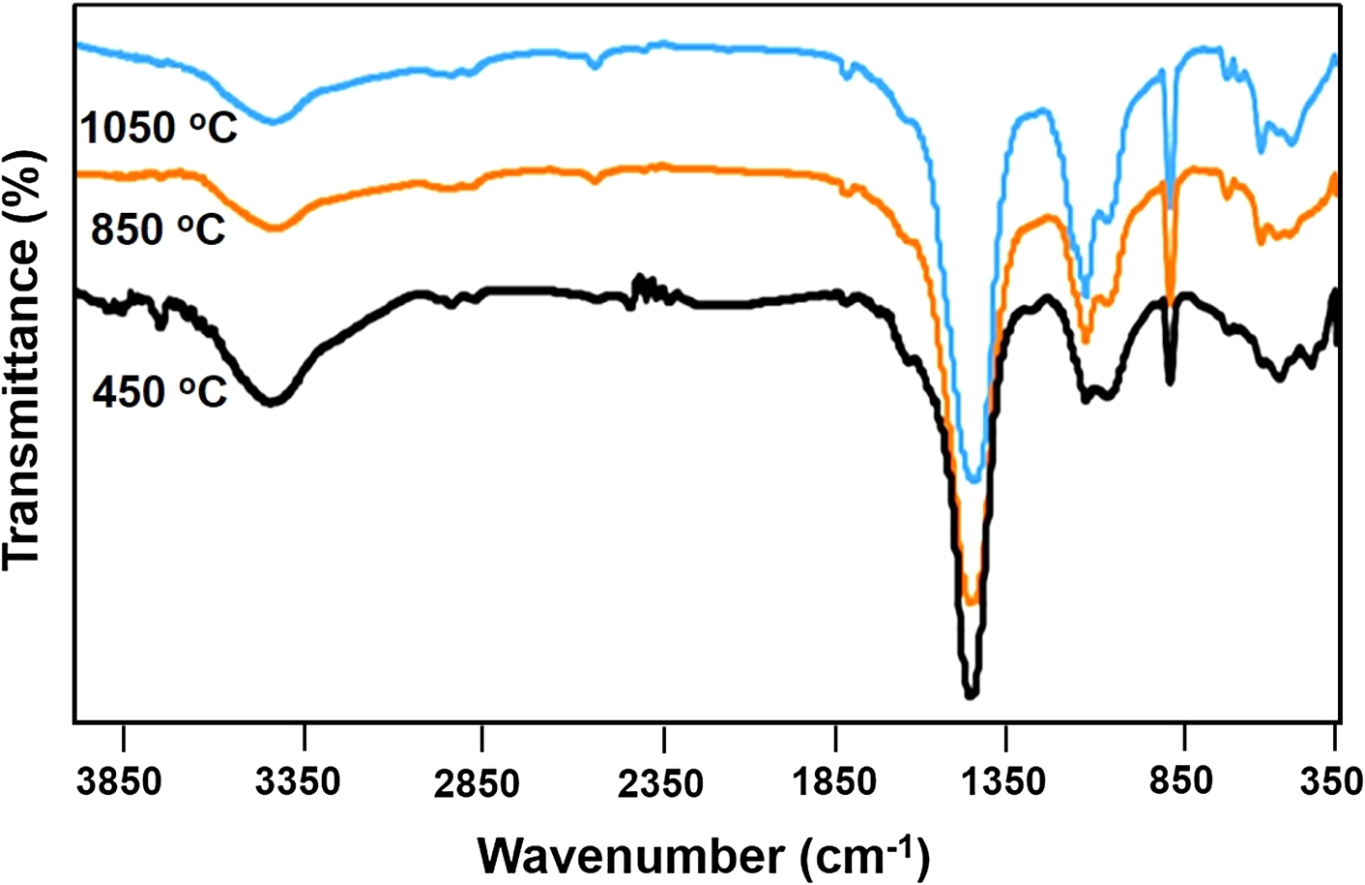
FT-IR spectra of RO WA prepared at different combustion temperatures.

The FT-IR spectra of burned WA samples at 450, 850, and 1050 °C provided in Fig. 4. The broad band at 3453 cm^−1^ belongs to hydroxyl stretching vibrations in several organic and inorganic constituents^37^, as can be seen in Fig. 4, the absorbance intensity decreases with increasing temperature due to burning of organic substances. The week absorbance bands at 2922 and 2853 cm^−1^ from C-H stretching vibrations are corresponded to aliphatic hydrocarbons in the WA and also the absorbance band at 1789 cm^−1^ and a shoulder peak at 1621 cm^−1^ belong to carbonyl and C=C groups, respectively^38^. The absorbance around 1793, 1443, 876, and 712 cm^−1^ are associated with carbonate (CO_3_^2−^), and also the characteristic bands at 1111, 1043, and 615 cm^−1^ for PO_4_^3−^ and SiO_2_ components, showing metal carbonate like CaCO_3_, SiO_2_ and metal phosphate. The FT-IR spectrum of WA burned at higher temperatures (ROA_850_ and ROA_1050_) show the absorbance bands at 1413, 1053, 523, and 471 cm^−1^, indicating presence of Si–O–Al, Si–O–Si and CaO functionalities^39^.

### Catalytic activity of WEWA in synthesis of benzochromene derivatives

**Figure 5:**
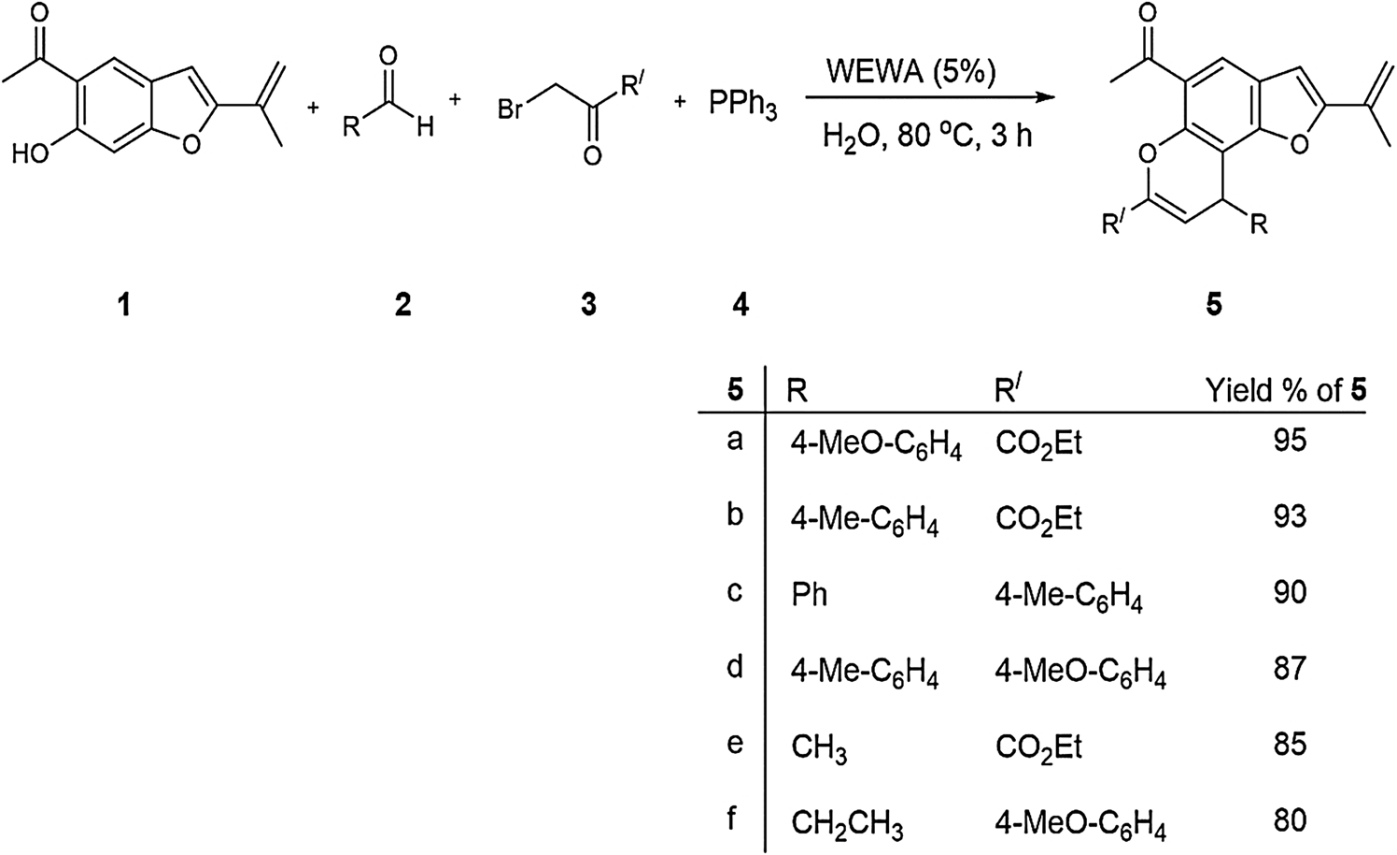
Three-component synthesis of benzochromene derivatives of **5** in water.

In this research work, we studied a green method for the preparation of some benzochromene derivatives by an efficient three component reaction of euparin **1**, aldehydes **2**, alkyl bromides **3** and triphenylphosphine **4** in the presence of a catalytic amounts of ROA_850_ (%5) in water at 80 °C with high yields (Fig. 5).

In the starting step of this work, condensation reaction of euparin** 1**^33^, 4-methoxy benzaldehyde **2**, ethyl bromopyruvate **3** and triphenylphosphine **4** at 80 °C in water was applied as a model reaction to obtain the optimum reaction conditions (Table 3).

**Table 3:** Effect of catalyst, its loading, and temperature on the condensation reaction of compound **5a**.

Entry	Catalyst	Temp. (°C)	Time (h)	Yield%^a^
1	None	Rt	15	–
2	None	70	15	Trace
3	None	90	15	Trace
4	ROA_850_ (%1)	70	2	55
**5**	**ROA**_**850**_** (%5)**	**70**	**3**	**99**
6	ROA_850_ (%10)	70	3	99
7	ROA_850_ (%5)	90	3	98
8	NaOH (%5)	70	5	48
9	NaOH (%10)	70	5	52
10	NaOH (%20)	70	5	51

^a^Isolated yield

These reactions did not progress without any catalyst even after 15 h (Table 3, entry 1). By increasing the reaction temperature to 70 and 90 °C, a trace amount of **5a** generated after 15 h (Table 3, entries 2 and 3). To acquire better results, ROA_850_ (%1) as a catalyst was added into the reaction mixture. Interestingly, 55% yield of **5a** was produced after 2 h (Table 3, entry 4). Then, the reaction was performed in the presence of ROA_850_ (%5). As it was expected, the yield of product **5a** was accomplished in 99% after 3 h under these reaction conditions (Table 3, entry 5). Consequently, various amounts of WEWA catalyst were utilized to discover the optimal catalyst loading. The results displayed that 5% of WEWA (ROA_850_) are enough for producing an excellent yield of **5a** (Table 3, entry 5). To clearly evaluate the catalytic activity of WA as a base catalyst, different percentages of NaOH were used in this reaction. Consequently, these results confirmed the main function of WA as the effective catalyst in this reaction. According to the optimized reaction conditions (Table 3), ROA_850_ (%5) as the catalyst in water at 70 °C was estimated to be the optimum amount of the catalyst for this reaction.

**Figure 6:**
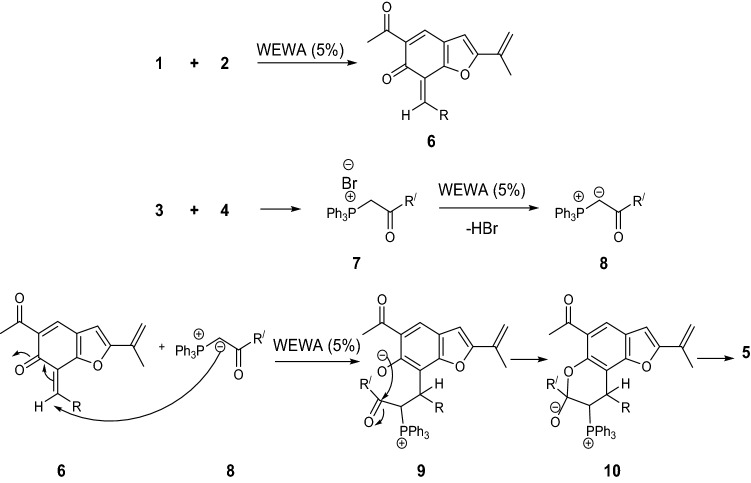
Proposed mechanism for the formation of **5**.

The structures of compounds **5** were verified by FT-IR, ^1^H NMR, ^13^C NMR, and mass spectral data (for detain see supporting information). For instance, the ^1^H NMR spectrum of **5a** revealed two singlets at δ = 2.15 and 2.52 ppm for methyl protons, four singlets at 4.58, 5.37, 6.14 and 7.75 ppm for methine proton along with signals for aromatic moiety. In the ^13^C NMR spectrum, the signals of the carbonyl group of **5a** were observed at δ 160.2 and 197.6 ppm. Although there is no exact information to approve the mechanistic details, it can be proposed as shown in Fig. 6.

First, euparin **1** and aldehyde **2** react in the presence of ROA_850_ (%5) that is generated intermediate **6**. In other reaction vials, triphenylphosphine and alkyl bromides reacted in the presence of ROA_850_ (%5) to produce the intermediate **8** by the elimination of HBr. The intermediate **8** attacks the intermediate **6** and produces intermediate **9**. The elimination of triphenylphosphine oxide and Cyclization of intermediate **9** provide compound **5**.

Alkali, alkaline metal, and metal oxides (e.g., Al_2_O_3_, CaO, MgO, Fe_2_O_3_, and SiO_2_) are widely used as both heterogeneous catalyst and catalyst support^40^. WA, a rich source of the aforementioned metal oxides, is an appropriate candidate for the reactions requiring basic catalysts. In addition, WA has a good catalytic activity and can be used as a solid base catalyst, and WA due to presence of some metal oxides such as CuO, Al_2_O_3_, SiO_2_, and CaO which increases the electrophilic activity of the carbonyl groups can increase the nucleophilic attack in the reaction media. Therefore, we expect that with the preparation of some benzochromene derivatives, both the basic power and nucleophilic activity will increase by WA.

**Figure 7:**
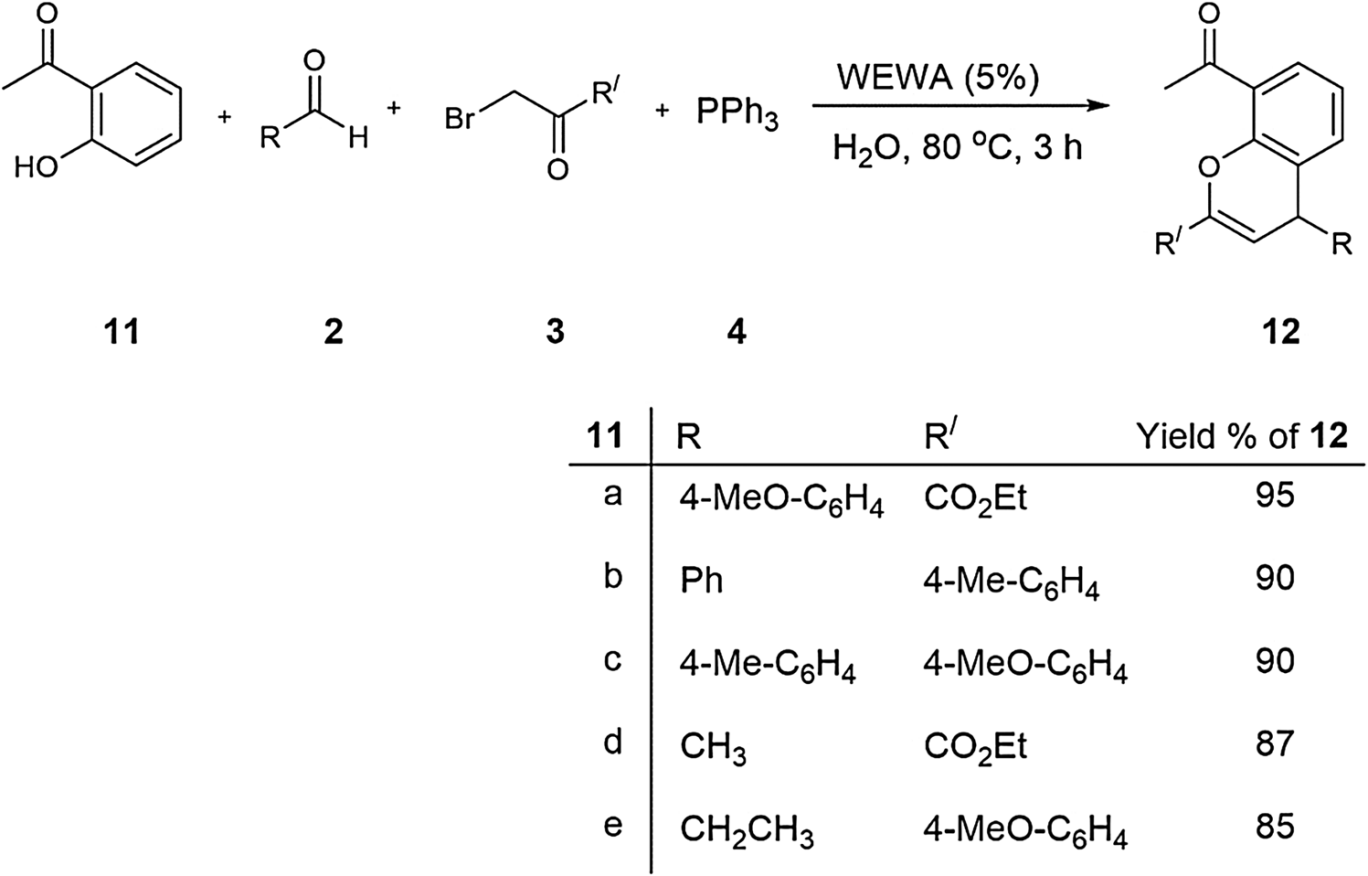
Three-component reaction for synthesis of benzochromene derivatives of **12** in water.

On the other hand, the main advantages of this procedure are green reaction conditions, economical procedure, utilization of small amounts of catalyst, high yield, short reaction times, and easy work-up, which are the required principles of green chemistry^41–45^. Under similar conditions, we also investigated the reaction between 2-hydroxyacetophenone **11**, aldehyde **2**, alkyl bromide **3** and triphenylphosphine **4** in the presence of ROA_850_ (%5) catalyst in water at 80 °C for confirming diversity of these reactions (Fig. 7).

## Experimental

### Materials and reagents

Three wood samples used for WA preparation in this study, pine (*Pinus alba*), poplar (*Populus nigra*) and Russian olive (*Elaeagnus angustifolia*), were collected from Mazandaran province, Iran. All the wood samples were dried prior to catalyst preparation. The chemicals and solvents used in this work were obtained from Sigma Aldrich. Euparin was extracted from *Petasites hybridus* dried roots according to our previous research^29,33^. All aqueous solutions are freshly prepared using distilled water. FT-IR spectra were recorded using pressed KBr disks, using Perkin–Elmer 781 spectrophotometer. X-ray diffraction (XRD) analyses were carried out with a Philips powder diffractometer type PW 1373 goniometer. The X-ray wavelength (1.5405 Å) and the diffraction patterns were recorded in the 2 h range (10–80°) with scanning rate of 2 °C/min. Fresh WA analyzed for elemental composition using XRF using a Philips 1404 wavelength dispersive spectrometer. All the samples were dried in an oven at 50 °C for 12 h to remove water content prior to the analyses. The morphology and particle dispersion were studied by SEM (Cam scan MV2300). The chemical composition of the prepared WA was confirmed by energy dispersive X-ray spectroscopy (EDS). The elemental analysis was employed to ascertain the resistance of C, H, and N using a Heraeus CHNO-Rapid analyzer. The mass spectra were collected using a FINNIGAN-MAT 8430 spectrometer operating at an ionization potential of 70 eV. FT-IR spectra were recorded on a Shimadzu IR-460 spectrometer. The ^1^H and ^13^C NMR spectra were analyzed using a Bruker DRX-500 advance spectrometer at 500.1 MHz and 125.8 MHz, respectively. The ^1^H and ^13^C spectra were performed for CDCl_3_ solutions applying TMS (as the internal standard) or 85 mass% H_3_PO_4_ (as external standard); chemical shifts (d) are given as parts per million (ppm).

### Preparation of WEWA

The fresh wood samples were washed properly with deionized water, cut into small pieces, and dried under the sunlight at open atmosphere until the constant weight. The dried samples were combusted at the rate of 20 °C/min to reach 450, 650, 850 and 1050 °C in the high temperature laboratory furnace under air condition and kept at the target temperatures for 4 h to form WA, which the amount of obtained ash for 1 kg of the wood samples (pine, poplar, and Russian olive) in different burning temperatures are presented in Table 1. The prepared WA samples indicated as WA_450_, WA_650_, WA_850_ and WA_1050_, where number 450 shows WA_450_ for WA burning at 450 °C and 650–1050 denotes the temperature of burning. Then 5 g of WA suspended in 100 mL deionized water in a beaker and stirred for 30 min at room temperature. The mixture filtered through a sintered glass container and the filtrate used as WEWA (5%) as a catalyst for some benzochromene derivatives.

### pH determination of WA for basicity measurement

0.5 g of different wood ash samples were suspended in 10 ml of deionized water and agitated for 24 h. The pH of suspension was measured using pH Meter. This measurement was performed for all of WA samples prepared at different temperatures as well as for various types of catalysts prepared from Russian olive ash.

### General procedure for preparation of compounds** 5a**–**f**

A mixture of 1-(6-hydroxy-2-isopropenyl-1-benzofuran-yl)-1-ethanone **1 **(2 mmol), aldehyde **2** (2 mmol) and 4 ml of WEWA catalyst (5%) was added to the magnetically stirred mixture of 4 ml of WEWA catalyst (5%), alkyl bromides **3** (2 mmol), and triphenylphosphine **4** at 80 °C. After completion the reaction by TLC monitoring, the catalyst was isolated by filtration, then the product was dissolved in ethyl acetate and purified by small column chromatography (CC) or paper chromatography (PC) (Hexane: EtOAc = 5:1). During the separation by CC or PC, remaining catalyst and triphenyl phosphine oxide were also removed from the product easily and finally washed with water to afford pure title compound **5**.

#### Ethyl 5-acetyl-2-isopropenyl-9-(4-methoxyphenyl)-9H-furo[2,3-f] chromene-7-carboxylate (**5a**)

Yellow powder, mp 163–165 °C, Yield: 0.82 g (95%). IR (KBr) (ν_max_/cm^−1^): 1724, 1738, 1675, 1585, 1462, 1274 cm^−1^. ^1^H NMR (500 MHz, CDCl_3_): 1.32 (3 H, t, ^3^*J* = 7.4 Hz, CH_3_), 2.15 (3 H, s, Me), 2.52 (3 H, s, Me), 3.75 (3 H, s, MeO), 4.26 (2 H, q, ^3^*J* = 7.3 Hz, CH_2_O), 4.58 (1 H, s, CH), 4.62 (1 H, d, ^2^*J* = 3.8 Hz, CH), 5.37 (1 H, s, CH), 5.68 (1 H, d, ^2^*J* = 3.8 Hz, CH), 6.14 (1 H, s, CH), 7.12 (2 H, d, ^3^*J* = 7.6 Hz, 2 CH), 7.63 (2 H, d, ^3^*J* = 7.6 Hz, 2 CH), 7.75 (1 H, s, CH) ppm. ^13^C NMR (125.7 MHz, CDCl_3_): 18.7 (Me), 22.6 (Me), 30.2 (Me), 41.2 (CH), 55.7 (MeO), 61.4 (CH_2_O), 109.3 (CH), 113.8 (2 CH), 114.3 (CH_2_), 117.4 (C), 117.8 (C), 120.5 (C), 122.6 (CH), 127.5 (CH), 128.6 (C), 131.7 (2 CH), 139.2 (C), 145.3 (C), 151.5 (C), 154.2 (C), 159.2 (C), 159.8 (C), 160.2 (C=O), 197.6 (C=O) ppm. EI-MS: 432 (M^+^, 15), 389 (86), 43 (100). Anal. Calcd for C_26_H_24_O_6_ (432.46): C 72.21, H 5.59; Found: C 72.36, H 5.72.

#### Ethyl 5-acetyl-2-isopropenyl-9-(4-methylphenyl)-9H-furo[2,3-f] chromene-7-carboxylate (**5b**)

Yellow powder, mp 158–160 °C, Yield: 0.82 g (93%). IR (KBr) (ν_max_/cm^−1^): 1726, 1742, 1683, 1575, 1472, 1295 cm^−1^. ^1^H NMR (500 MHz, CDCl_3_): 1.34 (3 H, t, ^3^*J* = 7.4 Hz, CH_3_), 2.16 (3 H, s, Me), 2.23 (3 H, s, Me), 2.53 (3 H, s, Me), 4.25 (2 H, q, ^3^*J* = 7.4 Hz, CH_2_O), 4.63 (1 H, s, CH), 4.65 (1 H, d, ^2^*J* = 4.2 Hz, CH), 5.45 (1 H, s, CH), 5.73 (1 H, d, ^2^*J* = 4.2 Hz, CH), 6.17 (1 H, s, CH), 7.32 (2 H, d, ^3^*J* = 7.6 Hz, 2 CH), 7.58 (2 H, d, ^3^*J* = 7.6 Hz, 2 CH), 7.78 (1 H, s, CH) ppm. ^13^C NMR (125.7 MHz, CDCl_3_): 18.5 (Me), 22.0 (Me), 22.7 (Me), 30.4 (Me), 40.8 (CH), 61.6 (CH_2_O), 109.5 (CH), 114.2 (CH_2_), 117.6 (C), 118.3(C), 120.5 (C), 122.8 (CH), 127.3 (CH), 128.5 (2 CH), 130.6 (2 CH), 136.4 (C), 139.2 (C), 145.2 (C), 151.6 (C), 154.5 (C), 159.5 (C), 160.4 (C=O), 197.4 (C=O) ppm. Anal. Calcd for C_26_H_24_O_5_ (416.47): C 74.98, H 5.81; Found: C 75.16, H 5.96.

#### 1-[2-isopropenyl-7-(4-methylphenyl)-9-phenyl-9H-furo[2,3-f] chromene-5-yl]-1-ethanone (**5c**)

Yellow powder, mp 172–174 °C, Yield: 0.76 g (90%). IR (KBr) (ν_max_/cm^−1^): 1725, 1692, 1563, 1485, 1274 cm^−1^. ^1^H NMR (500 MHz, CDCl_3_): 2.15 (3 H, s, Me), 2.35 (3 H, s, Me), 2.54 (3 H, s, Me), 4.72 (1 H, s, CH), 4.74 (1 H, d, ^2^*J* = 4.5 Hz, CH), 5.04 (1 H, s, CH), 5.53 (1 H, s, CH), 5.76 (1 H, d, ^2^*J* = 4.5 Hz, CH), 6.75 (1 H, t, ^3^*J* = 7.5 Hz, CH), 7.12 (2 H, t, ^3^*J* = 7.5 Hz, 2 CH), 7.35 (2 H, d, ^3^*J* = 7.8 Hz, 2 CH), 7.46 (2 H, d, ^3^*J* = 7.5 Hz, 2 CH), 7.73 (2 H, d, ^3^*J* = 7.8 Hz, 2 CH), 7.82 (1 H, s, CH) ppm. ^13^C NMR (125.7 MHz, CDCl_3_): 18.6 (Me), 21.6 (Me), 30.5 (Me), 42.3 (CH), 102.4 (CH), 109.3 (CH), 114.4 (CH_2_), 117.8 (C), 118.4 (C), 120.6 (C), 122.8 (CH), 125.6 (2 CH), 126.7 (CH), 127.3 (2 CH), 128.4 (2 CH), 129.4 (2 CH), 133.2 (C), 135.3 (C), 137.5 (C), 139.4 (C), 152.6 (C), 153.3 (C), 153.8 (C), 159.3 (C), 197.5 (C=O) ppm. Anal. Calcd for C_29_H_24_O_3_ (420.49): C 82.83, H 5.75; Found: C 82.96, H 5.92.

#### 1-[2-isopropenyl-7-(4-methoxyphenyl)-9-(4-methylphenyl)-9H-furo[2,3-f] chromene-5-yl]-1-ethanone (**5d**)

Yellow powder, mp 183–185 °C, Yield: 0.78 g (87%). IR (KBr) (ν_max_/cm^−1^): 1726, 1689, 1572, 1486, 1282 cm^−1^. ^1^H NMR (500 MHz, CDCl_3_): 2.12 (3 H, s, Me), 2.17 (3 H, s, Me), 2.56 (3 H, s, Me), 3.87 (3 H, s, MeO), 4.75 (1 H, s, CH), 4.82 (1 H, d, ^2^*J* = 3.6 Hz, CH), 5.12 (1 H, s, CH), 5.62 (1 H, s, CH), 5.83 (1 H, d, ^2^*J* = 3.6 Hz, CH), 7.32 (2 H, d, ^3^*J* = 7.6 Hz, 2 CH), 7.38 (2 H, d, ^3^*J* = 7.6 Hz, 2 CH), 7.62 (2 H, d, ^3^*J* = 7.6 Hz, 2 CH), 7.75 (2 H, d, ^3^*J* = 7.6 Hz, 2 CH), 7.85 (1 H, s, CH) ppm. ^13^C NMR (125.7 MHz, CDCl_3_): 18.7 (Me), 21.8 (Me), 30.6 (Me), 42.4 (CH), 55.6 (MeO), 102.3 (CH), 109.5 (CH), 114.5 (CH_2_), 114.9 (2 CH), 117.6 (C), 118.6 (C), 120.7 (C), 122.7 (CH), 127.6 (2 CH), 128.5 (2 CH), 129.0 (C), 129.5 (2 CH), 130.4 (C), 136.2 (C), 139.3 (C), 152.7 (C), 153.3 (C), 153.8 (C), 159.3 (C), 161.6 (C), 198.2 (C=O) ppm. Anal. Calcd for C_30_H_26_O_4_ (450.53): C 79.98, H 5.82; Found: C 80.16, H 5.97.

#### Ethyl 5-acetyl-2-isopropenyl-9-methyl-9H-furo[2,3-f] chromene-7-carboxylate (**5e**)

Yellow powder, mp 131–133 °C, Yield: 0.58 g (85%). IR (KBr) (ν_max_/cm^−1^): 1723, 1742, 1684, 1587, 1468, 1275 cm^−1^. ^1^H NMR (500 MHz, CDCl_3_): 1.33 (3 H, t, ^3^*J* = 7.4 Hz, CH_3_), 1.54 (3 H, d, ^3^*J* = 7.4 Hz, CH_3_), 2.16 (3 H, s, Me), 2.54 (3 H, s, Me), 3.94 (1 H, q, ^3^*J* = 7.4 Hz, CH), 4.25 (2 H, q, ^3^*J* = 7.4 Hz, CH_2_O), 4.65 (1 H, d, ^2^*J* = 4.0 Hz, CH), 5.42 (1 H, s, CH), 5.73 (1 H, d, ^2^*J* = 4.0 Hz, CH), 6.05 (1 H, s, CH), 7.83 (1 H, s, CH) ppm. ^13^C NMR (125.7 MHz, CDCl_3_): 18.5 (Me), 20.4 (Me), 22.6 (Me), 30.4 (Me), 31.0 (CH), 61.5 (CH_2_O), 108.6 (CH), 114.2 (CH_2_), 116.8 (C), 117.6 (C), 120.7 (C), 122.7 (CH), 128.6 (CH), 136.5 (C), 143.8 (C), 151.6 (C), 154.2 (C), 158.6 (C), 160.3 (C=O), 198.3 (C=O) ppm. Anal. Calcd for C_20_H_20_O_5_ (340.37): C 70.57, H 5.92; Found: C 70.68, H 6.04.

#### 1-[9-ethyl-2-isopropenyl-7-(4-methoxyphenyl)-9H-furo [2,3-f] chromene-5-yl]-1-ethanone (**5f**)

Yellow powder, mp 131–133 °C, Yield: 0.62 g (80%). IR (KBr) (ν_max_/cm^−1^): 1727, 1693, 1590, 1485, 1283 cm^−1^. ^1^H NMR (500 MHz, CDCl_3_): 1.12 (3 H, t, ^3^*J* = 7.2 Hz, CH_3_), 1.48–1.57 (1 H, m, CH), 1.65–1.78 (1 H, m, CH), 2.14 (3 H, s, Me), 2.56 (3 H, s, Me), 3.79–3.87 (1 H, m, CH), 3.91(3 H, s, MeO), 4.74 (1 H, d, ^2^*J* = 4.2 Hz, CH), 5.48 (1 H, s, CH), 5.75 (1 H, d, ^2^*J* = 4.2 Hz, CH), 6.12 (1 H, s, CH), 7.28 (2 H, d, ^3^*J* = 7.6 Hz, 2 CH), 7.65 (2 H, d, ^3^*J* = 7.6 Hz, 2 CH), 7.87 (1 H, s, CH) ppm. ^13^C NMR (125.7 MHz, CDCl_3_): 13.2 (CH_3_), 18.6 (Me), 29.6 (CH_2_), 30.5 (Me), 31.2 (CH), 55.6 (MeO), 103.5 (CH), 107.6 (CH), 113.8 (2 CH), 114.5 (CH_2_), 116.7 (C), 117.5 (C), 119.8 (C), 122.3 (CH), 127.6 (2 CH), 129.6 (C), 135.4 (C), 151.7 (C), 154.3 (C), 155.2 (C), 157.4 (C), 161.9 (C=O), 197.2 (C=O) ppm. EI-MS: 388 (M^+^, 15), 345 (68), 43 (100). Anal. Calcd for C_25_H_24_O_4_ (388.46): C 77.30, H 6.23; Found: C 77.46, H 6.38.

### General procedure for the preparation of compounds **12**

A mixture of 2-hydroxyacetophenone **11** (2 mmol), aldehyde **2** (2 mmol) and 4 ml of WEWA catalyst (5%) added to the magnetically stirred mixture of 4 ml of WEWA catalyst (5%), alkyl bromides **3** (2 mmol), triphenylphosphine **4** (2 mmol) at 80 °C. After completion of the reaction by TLC monitoring, the catalyst was isolated by filtration, then the product was dissolved in ethyl acetate and purified by small CC or PC (Hexane: EtOAc = 5:1). During the separation, the remaining catalyst and triphenylphosphine oxide were removed from the product and finally it was washed with water to attain pure title compound **12**.

#### Ethyl 8-acetyl-4-(4-methoxyphenyl)-4H-chromene-2-carboxylate (**12a**)

Yellow powder, mp 103–105 °C, Yield: 0.65 g (93%). IR (KBr) (ν_max_/cm^−1^): 1725, 1743, 1692, 1595, 1484, 1268 cm^−1^. ^1^H NMR (500 MHz, CDCl_3_): 1.28 (3 H, t, ^3^*J* = 7.4 Hz, CH_3_), 2.53 (3 H, s, Me), 3.85 (3 H, s, MeO), 4.25 (2 H, q, ^3^*J* = 7.4 Hz, CH_2_O), 4.46 (1 H, s, CH), 6.34 (1 H, s, CH), 6.95 (2 H, d, ^3^*J* = 7.6 Hz, 2 CH), 7.18 (1 H, t, ^3^*J* = 7.8 Hz, CH), 7.42 (1 H, d, ^3^*J* = 7.8 Hz, CH), 7.52 (2 H, d, ^3^*J* = 7.6 Hz, 2 CH), 7.95 (1 H, d, ^3^*J* = 7.8 Hz, CH) ppm. ^13^C NMR (125.7 MHz, CDCl_3_): 22.5 (Me), 30.6 (Me), 45.8 (CH), 55.6 (MeO), 62.3 (CH_2_O), 114.3 (2 CH), 124.2 (CH), 126.4 (CH), 129.7 (2 CH), 130.6 (C), 131.2 (CH), 131.8 (CH), 132.3 (CH), 145.2 (C), 154.2 (C), 159.2 (C), 160.3 (C=O), 198.3 (C=O) ppm. Anal. Calcd for C_21_H_20_O_5_ (352.38): C 71.58, H 5.72; Found: C 71.73, H 5.87.

#### 1-[2-(4-methylphenyl)-4-phenyl-4H-chromene-8-yl]-1-ethanone (**12b**)

Yellow powder, mp 123–125 °C, Yield: 0.59 g (87%). IR (KBr) (ν_max_/cm^−1^): 1727, 1687, 1576, 1487, 1282 cm^−1^. ^1^H NMR (500 MHz, CDCl_3_): 2.36 (3 H, s, Me), 2.62 (3 H, s, Me), 4.52 (1 H, s, CH), 5.35 (1 H, s, CH), 6.35 (1 H, t, ^3^*J* = 7.5 Hz, CH), 7.04 (2 H, t, ^3^*J* = 7.5 Hz, 2 CH), 7.15 (1 H, t, ^3^*J* = 7.6 Hz, CH), 7.35 (2 H, d, ^3^*J* = 7.6 Hz, 2 CH), 7.42 (1 H, d, ^3^*J* = 7.5 Hz, CH), 7.53 (2 H, d, ^3^*J* = 7.6 Hz, 2 CH), 7.76 (2 H, d, ^3^*J* = 7.6 Hz, 2 CH), 8.03 (1 H, d, ^3^*J* = 7.5 Hz, CH) ppm. ^13^C NMR (125.7 MHz, CDCl_3_): 21.5 (Me), 30.7 (Me), 47.3 (CH), 101.4 (CH), 124.2 (CH), 124.8 (C), 125.8 (2 CH), 127.3 (CH), 128.0 (2 CH), 128.4 (2 CH), 129.1 (2 CH), 131.2 (CH), 131.8 (CH), 132.2 (C), 135.3 (C), 137.2 (C), 139.7 (C), 153.4 (C), 155.7 (C), 198.6 (C=O) ppm. Anal. Calcd for C_24_H_20_O_2_ (340.41): C 84.68, H 5.92; Found: C 84.82, H 6.12.

#### 1-[2-(4-methoxyphenyl)-4-(4-methylphenyl)-4H-chromene-8-yl]-1-ethanone (**12c**)

Yellow powder, mp 127–129 °C, Yield: 0.63 g (85%). IR (KBr) (ν_max_/cm^−1^): 1728, 1694, 1586, 1485, 1275 cm^−1^. ^1^H NMR (500 MHz, CDCl_3_): 2.14 (3 H, s, Me), 2.58 (3 H, s, Me), 3.85 (3 H, s, MeO), 4.56 (1 H, s, CH), 5.36 (1 H, s, CH), 7.18 (2 H, d, ^3^*J* = 7.6 Hz, 2 CH), 7.24 (1 H, t, ^3^*J* = 7.5 Hz, CH), 7.32 (2 H, d, ^3^*J* = 7.6 Hz, 2 CH), 7.42 (1 H, d, ^3^*J* = 7.5 Hz, CH), 7.58 (2 H, d, ^3^*J* = 7.6 Hz, 2 CH), 7.75 (2 H, d, ^3^*J* = 7.6 Hz, 2 CH), 7.96 (1 H, d, ^3^*J* = 7.5 Hz, CH) ppm. ^13^C NMR (125.7 MHz, CDCl_3_): 21.7 (Me), 30.8 (Me), 47.4 (CH), 55.6 (MeO), 101.6 (CH), 114.6 (2 CH), 124.3 (CH), 126.4 (2 CH), 127.8 (2 CH), 130.2 (2 CH), 130.6 (C), 131.4 (CH), 131.8 (CH), 132.2 (C), 136.2 (C), 153.2 (C), 155.6 (C), 161.4 (C), 198.5 (C=O) ppm. Anal. Calcd for C_25_H_22_O_3_ (370.44): C 81.06, H 5.99; Found: C 81.21, H 6.14.

#### Ethyl 8-acetyl-4-methyl-4H-chromene-2-carboxylate (**12d**)

Yellow powder, mp 100–102 °C, Yield: 0.42 g (80%). IR (KBr) (ν_max_/cm^−1^): 1725, 1738, 1687, 1562, 1473, 1295 cm^−1^. ^1^H NMR (500 MHz, CDCl_3_): 1.35 (3 H, t, ^3^*J* = 7.4 Hz, CH_3_), 1.43 (3 H, d, ^3^*J* = 7.3 Hz, CH_3_), 2.58 (3 H, s, Me), 3.92 (1 H, q, ^3^*J* = 7.3 Hz, CH), 4.26 (2 H, q, ^3^*J* = 7.4 Hz, CH_2_O), 5.86 (1 H, s, CH), 7.12 (1 H, t, ^3^*J* = 7.5 Hz, CH), 7.32 (1 H, d, ^3^*J* = 7.6 Hz, CH), 7.93 (1 H, d, ^3^*J* = 7.6 Hz, CH) ppm. ^13^C NMR (125.7 MHz, CDCl_3_): 19.2 (Me), 22.8 (Me), 30.6 (Me), 35.4 (CH), 61.4 (CH_2_O), 123.6 (C), 124.2 (CH), 127.6 (CH), 130.5 (C), 131.2 (CH), 131.8 (CH), 143.6 (C), 154.3 (C), 160.5 (C=O), 198.5 (C=O) ppm. Anal. Calcd for C_15_H_16_O_4_ (260.28): C 69.22, H 6.20; Found: C 69.36, H 6.34.

#### 1-[2-ethyl-4-(4-methoxyphenyl)-4H-chromene-8-yl]-1-ethanone (**12e**)

Yellow powder, mp 118–120 °C, Yield: 0.51 g (83%). IR (KBr) (ν_max_/cm^−1^): 1726, 1683, 1587, 1464, 1275 cm^−1^. ^1^H NMR (500 MHz, CDCl_3_): 1.15 (3 H, t, ^3^*J* = 7.3 Hz, CH_3_), 1.45–1.54 (1 H, m, CH), 1.67–1.82 (1 H, m, CH), 2.58 (3 H, s, Me), 3.76–3.84 (1 H, m, CH), 3.87 (3 H, s, MeO), 5.83 (1 H, s, CH), 7.04 (1 H, t, ^3^*J* = 7.4 Hz, CH), 7.27 (2 H, d, ^3^*J* = 7.6 Hz, 2 CH), 7.56 (1 H, d, ^3^*J* = 7.5 Hz, CH), 7.65 (2 H, d, ^3^*J* = 7.6 Hz, 2 CH), 7.86 (1 H, d, ^3^*J* = 7.5 Hz, CH) ppm. ^13^C NMR (125.7 MHz, CDCl_3_): 13.4 (CH_3_), 29.3 (CH_2_), 30.2 (Me), 35.6 (CH), 55.7 (MeO), 102.4 (CH), 114.2 (2 CH), 123.7 (CH), 127.4 (2 CH), 129.7 (C), 130.3 (C), 131.0 (CH), 131.6 (CH), 151.8 (C), 156.8 (C), 160.7 (C), 198.6 (C=O) ppm. Anal. Calcd for C_20_H_20_O_3_ (308.37): C 77.90, H 6.54; Found: C 78.06, H 6.68.

### Conclusions

We have reported an efficient and facile approach for the synthesis of novel benzochromene derivatives via the multi components reaction of euparin with aldehydes, alkyl bromides, and using triphenylphosphine in the presence of water extracts of wood ash as a catalyst. To the best of our knowledge, this is the first report of this catalyst and its application for the synthesis of these important compounds. Advantages of the presented procedure are high to excellent yields, simple work-up and the lack of need for column chromatography (Supplementary Information).

## Supplementary Information

Supplementary Information.

## References

[1] Etiégni L, Campbell AG (1991). Physical and chemical characteristics of wood ash. Bioresour. Technol..

[2] Olanders B, Steenari B (1995). Characterization of ashes from wood and straw. Biomass Bioenergy.

[3] Aprianti SE (2017). A huge number of artificial waste material can be supplementary cementitious material (SCM) for concrete production—A review part II. J. Clean. Prod..

[4] Werkelin J, Skrifvars BJ, Hupa M (2005). Ash-forming elements in four. Scandinavian wood species. Part 1: Summer harvest. Biomass Bioenergy.

[5] Erich MS, Ohno T (1992). Titrimetric determination calcium carbonate equivalence of wood ash. Analyst.

[6] Ulery L, Graham RC, Amrhein C (1993). Wood-ash composition and soil pH following intense burning. Soil Sci..

[7] Gholipour B, Shojaei S, Rostamnia S, Naimi-Jamal MR, Kim D, Kavetskyy T, Nouruzi N, Jang HW, Varma RS, Shokouhimehr M (2021). Metal-free nanostructured catalysts: Sustainable driving forces for organic transformations. Green. Chem..

[8] Al-Rahbi S, Williams PT (2019). Waste ashes as catalysts for the pyrolysis–catalytic steam reforming of biomass for hydrogen-rich gas production. J. Mater. Cycles Waste Manag..

[9] Deyris PA, Adler P, Petit E, Legrand YM, Grison C (2019). Woody species: A new bio-based material for dual Ca/Mg catalysis with remarkable Lewis acidity properties. Green Chem..

[10] Miladinović MR, Zdujić MV, Veljović DN, Krstić JB, Banković-Ilić IB, Veljković VB, Stamenković OS (2020). Valorization of walnut shell ash as a catalyst for biodiesel production. Renew. Energy.

[11] Sharma M, Ali Khan A, Puri SK, Tuli DK (2012). Wood ash as a potential heterogeneous catalyst for biodiesel synthesis. Biomass Bioenergy.

[12] Lopinti K, Sharma M, Chakradhar M, Arora AK, Kagdiyal V, Majumdar SK (2020). Ash catalyzed synthesis of long-chain dialkyl carbonates through carbonyl exchange reaction. Catal. Lett..

[13] Basumatary S, Nath B, Kalita P (2018). Application of agro-waste derived materials as heterogeneous base catalysts for biodiesel synthesis. J. Renew. Sustain. Energy..

[14] Shokouhimehr M, Mahmoudi-Gom Yek S, Nasrollahzadeh M, Kim A, Varma RS (2019). Palladium nanocatalysts on hydroxyapatite: Green oxidation of alcohols and reduction of nitroarenes in water. Appl. Sci..

[15] Khafagy MM, Abd El-Wahab AHF, Eid FA, El-Agrody AM (2002). Synthesis of halogen derivatives of benzo[h]chromene and benzo[a]anthracene with promising antimicrobial activities. Farmaco.

[16] Sashidhara KV, Rosaiah JN, Bhatia G, Saxena JK (2008). Novel keto-enamine Schiffs bases from 7-hydroxy-4-methyl-2-oxo-2H-benzo[h] chromene-8,10-dicarbaldehyde as potential antidyslipidemic and antioxidant agents. Eur. J. Med. Chem..

[17] De Andrade-Neto VF, Goulart MOF, Da Silva Filho JF, Da Silva MJ, Pinto MDCFR, Pinto AV, Zalis MG, Carvalho LH, Krettli AU (2004). Antimalarial activity of phenazines from lapachol, β-lapachone and its derivatives against *Plasmodium falciparum* in vitro and *Plasmodium berghei* in vivo. Bioorg. Med. Chem. Lett..

[18] Kanakaraju S, Prasanna B, Basavoju S, Chandramouli GVP (2012). Ionic liquid catalyzed one-pot multi-component synthesis, characterization and antibacterial activity of novel chromeno [2, 3-d] pyrimidin-8-amine derivatives. J. Mol. Struct..

[19] Qiang Z, Shi JB, Song BA, Liu XH (2014). Novel 2H-chromen derivatives: Design, synthesis and anticancer activity. RSC Adv..

[20] Balou J, Khalilzadeh MA, Zareyee D (2019). An efficient and reusable nano catalyst for the synthesis of benzoxanthene and chromene derivatives. Sci. Rep..

[21] Yang F, Wang H, Jiang L, Yue H, Zhang H, Wang Z, Wang L (2015). A green and one-pot synthesis of benzo[g]chromene derivatives through a multi-component reaction catalyzed by lipase. RSC Adv..

[22] Maleki B, Babaee S, Tayebee R (2015). Zn (l-Proline)2: As a powerful and reusable organomettalic catalyst for the very fast synthesis of 2-amino-4H-benzo[g]chromene derivatives under solvent-free conditions. Appl. Organomet. Chem..

[23] Khurana JM, Nand B, Saluja P (2010). DBU: A highly efficient catalyst for one-pot synthesis of substituted 3,4-dihydropyrano[3,2-c]chromenes, dihydropyrano[4,3-b]pyranes, 2-amino-4H-benzo[h]chromenes and 2-amino-4H benzo[g]chromenes in aqueous medium. Tetrahedron.

[24] Yao C, Yu C, Li T, Tu S (1989). An efficient synthesis of 4H-benzo[g]chromene-5,10-dione de-rivatives through triethylbenzylammonium chloride catalyzed multicomponent reaction under solvent-free conditions. Chin. J. Chem..

[25] Yu Y, Guo H, Li X (2011). An improved procedure for the three-component synthesis of benzo[g]chromene derivatives using basic ionic liquid. J. Heterocycl. Chem..

[26] Mirosanloo A, Zareyee D, Khalilzadeh MA (2018). Recyclable cellulose nanocrystal supported Palladium nanoparticles as an efficient heterogeneous catalyst for the solvent-free synthesis of coumarin derivatives via von Pechmann condensation. Appl. Organomet. Chem..

[27] Rajabi M, Hossaini Z, Khalilzadeh MA, Datta S, Halder M, Mousa SA (2015). Synthesis of a new class of furo [3, 2-c] coumarins and its anticancer activity. J. Photochem. Photobiol. B..

[28] Yaghoobi M, Zareyee D, Khalilzadeh MA (2020). A green and chemoselective synthesis of coumarins via Pechmann condensation using recoverable heterogeneous catalyst (Au@pSiO_2_). Appl. Organomet. Chem..

[29] Khaleghi F, Bin Din L, Jantan J, Yaacob WA, Khalilzadeh MA (2011). A facile synthesis of novel 1,4-benzoxazepin-2-one derivatives. Tetrahedron Lett..

[30] Khalilzadeh MA, Hossaini Z, Charati FR, Hallajian S, Rajabi M (2011). A mild and efficient method for the synthesis of a new class of furo [3, 2-c] chromenes in aqueous media. Mol. Div..

[31] Charati FR, Hossaini Z, Khalilzadeh MA (2012). Novel isocyanide-based three-component synthesis of substituted 9Hfuro [2, 3-f] chromene-8, 9-dicarboxylates in water. Comb. Chem. High Throughput Screen..

[32] Hallajian S, Khalilzadeh MA, Tajbakhsh M, Alipour E, Safaei Z (2015). Nano clinoptilolite: Highly efficient catalyst for the synthesis of chromene derivatives under solvent-free conditions. Comb. Chem. High Throughput Screen..

[33] Khaleghi F, Bin Din L, Rostami Charati F, Yaacob WA, Khalilzadeh MA, Skelton B, Makha M (2011). A new bioactive compound from the roots of *Petasites hybridus*. Phytochem. Lett..

[34] Ulery L, Graham RC, Amrhein C (1993). Wood-ash composition and soil pH following intense burning. Soil Sci..

[35] Liodakis S, Katsigiannis G, Kakali G (2005). Ash properties of some dominant Greek forest species. Thermochim. Acta..

[36] Misra MK, Ragland KW, Baker AJ (1993). Wood ash composition as a function of furnace temperature. Biomass Bioenergy.

[37] Gerzabek MH, Antil RS, Kögel-Knabner I, Knicker H, Kirchmann H, Haberhauer G (2006). How are soil use and management reflected by soil organic matter characteristics: A spectroscopic approach. Eur. J. Soil Sci..

[38] Dick DP, Knicker H, Ávila LG, Inda AV, Giasson E, Bissani CA (2006). Organic matter in constructed soils from a coal mining area in southern Brazil. Org. Geochem..

[39] Radev L, Hristov V, Michailova I, Helena M, Fernandes V, Miranda I (2010). In vitro bioactivity of biphasic calcium phosphate silicate glass-ceramic in CaO-SiO_2_-P_2_O_5_ system. Process Appl. Ceram..

[40] Pitman RM (2006). Wood ash use in forestry—A review of the environmental impacts. Int. J. For. Res..

[41] Shokouhimehr M, Kim JH, Lee YS (2006). Heterogeneous Heck reaction catalyzed by recyclable polymer-supported N-heterocyclic carbene-palladium complex. Synlett.

[42] Anastas P, Eghbali N (2010). Green chemistry: Principles and practice. Chem. Soc. Rev..

[43] Shokouhimehr M (2015). Magnetically separable and sustainable nanostructured catalysts for heterogeneous reduction of nitroaromatics. Catalysts.

[44] Nayebi B, Rabiee N, Nayebi B, Shahedi Asl M, Ramakrishna S, Jang HW, Varma RS, Shokouhimehr M (2020). Boron nitride-palladium nanostructured catalyst: Efficient reduction of nitrobenzene derivatives in water. Nano Express.

[45] Zhang K, Suh JM, Lee TH, Cha JH, Choi JW, Jang HW, Varma RS, Shokouhimehr M (2019). Copper oxide–graphene oxide nanocomposite: Efficient catalyst for hydrogenation of nitroaromatics in water. Nano Convergence.

