# Supplementary material for: Wood ash biocatalyst as a novel green catalyst and its application for the synthesis of benzochromene derivatives
Source: Sci Rep. 2022 Jan 21;12:1145. doi: 10.1038/s41598-022-05133-x (PMC8782886; doi:10.1038/s41598-022-05133-x)
Supplement: Supplementary file 1 — Supplementary Information. [file 41598_2022_5133_MOESM1_ESM.docx]

**SUPPLEMENTARY MATERIAL**

**Wood ash biocatalyst as a novel green catalyst and its application for the synthesis of benzochromene derivatives**

Rezvaneh Rostamian, Mohammad A. Khalilzadeh^*^, Daryoush Zareyee

Department of Chemistry, Qaemshahr Branch, Islamic Azad University, Qaemshahr, Iran

*Corresponding author: Tel +98-911-0400; *E-mail address:* [khalilzadeh73@gmail.com](mailto:khalilzadeh73@gmail.com) (M. A. Khalilzadeh)

**1. Antioxidant activity evaluations**

**1.1. DPPH radical scavenging activity**

Diphenyl-2-picrylhydrazyl (DPPH) radical scavenging study is widely applied for the assessment of compounds to scavange free radicals and their antioxidant reactivity in biological systems and foods [S1-S2]. The DPPH analyze can determin the hydrogen atom activity (or one electron) and provide antioxidant activity because of the free radical capturing. The antioxidant activity of **5a, 5b, 12a** and **12b** were studied by testing their capability to the DPPH radical. DPPH radical presents the absorption in 517 nm, however, it decreases when is reduced by an antioxidant or a radical species. The antioxidant activity of **5a**, **5b**, **12a** and **12b** were compared with BHT and TBHQ at different concentrations (from 200 mmol/L to 1000 mmol/L). The new prepared compounds had significant differences compared to BHT and TBHQ in all the tested concentrationsAll the compounds showed superior free radical scavenging efficiency compared to BHT and TBHQ at the concentration of 1000 ppm (Figure S1).


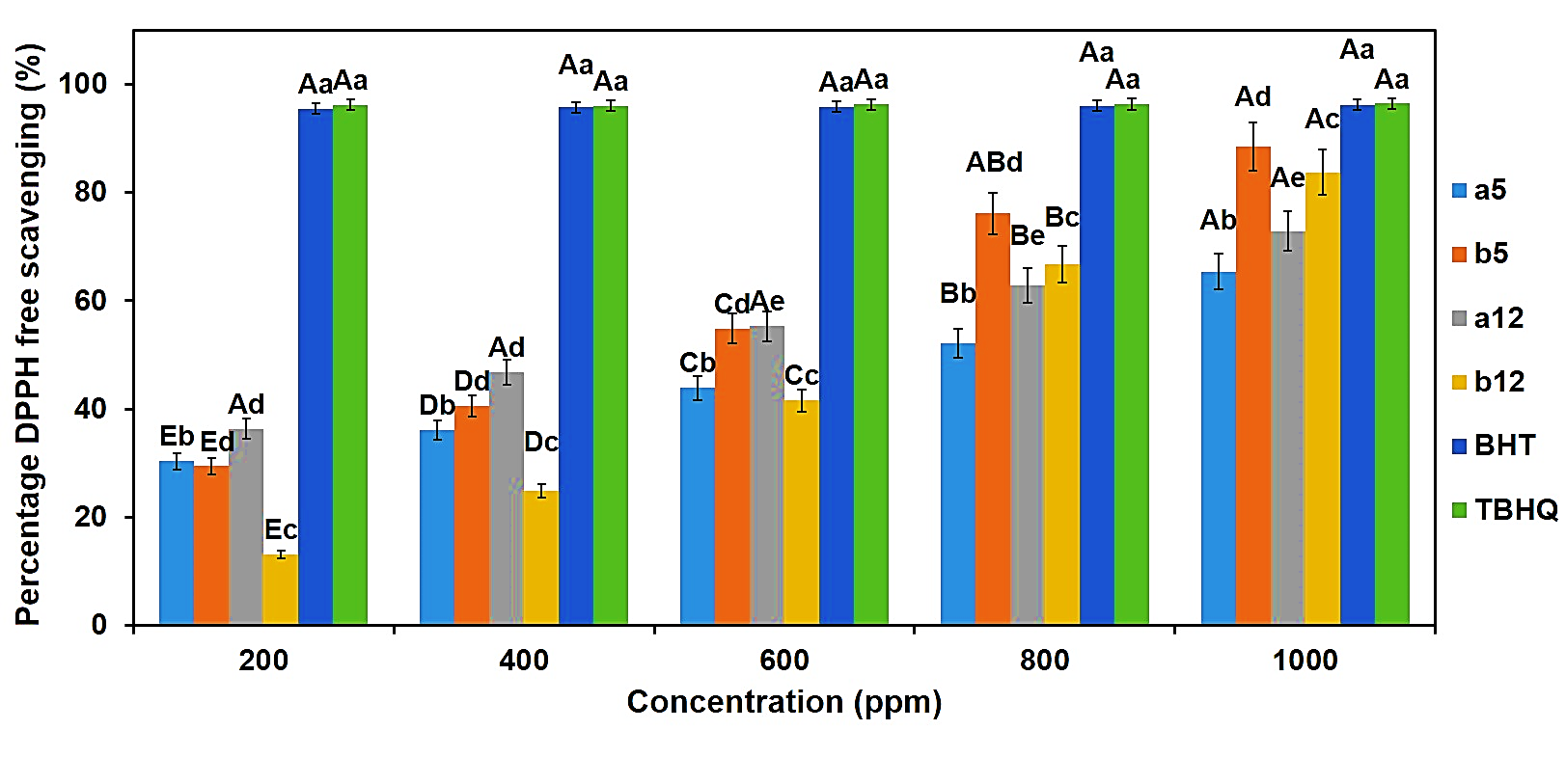


**Figure S1.** Radical scavenging activity of **5a**, **5b**, **12a** and **12b**. Differences between small letters of different samples in a concentration indicate significant differences (*P*≤ 0.05). Differences between capital letters in different concentrations of a sample indicate significant differences (*P*≤ 0.05).

**1.2. Ferric ions (Fe^3+^) reducing potential (FRAP)**

The susceptibility of the synthesized compounds to reduce Ferric ions (Fe^3+^) was invstigated by the measurement of the Fe^3+^/ferricyanide complex exchange to the Fe^2+^/ferrous shape at 700 nm [S1]. The capability of a compound to reducing may function as a significant indicator of its potential antioxidant activity. Compound **5a** and **5b** showed moderate reducing reactivity compared to standards (BHT and TBHQ); but, **12a** and **12b** had lower Fe^+3^ reducing potential than to **5a**, **5b**, BHT and TBHQ. It seems that the **5a** and **5b** had the 1-(4*H*-chromene-8-yl) ethanone core with higher iron chelating affinity than to **12a** and 1**2b** that lead to high reducing potential. The results are presented in Figure S2.


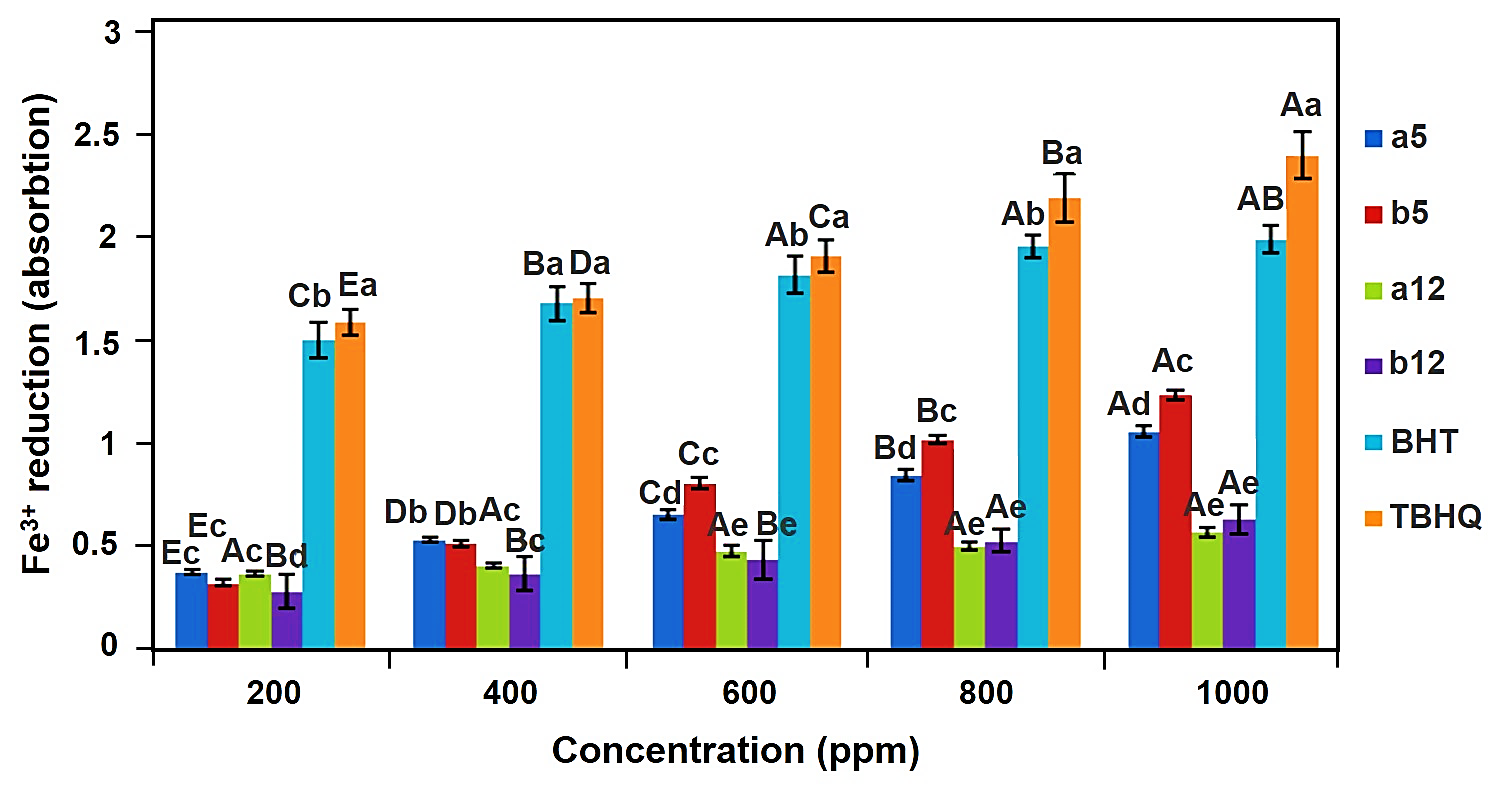


**Figure S2.** Fe^3+^ FRAP of compounds **5a, 5b, 12a** and **12b**. Differences between small letters of different samples in a concentration indicate significant differences (*P*≤ 0.05). Differences between capital letters in different concentrations of a sample indicate significant differences (*P*≤ 0.05).

**2.** **Experimental**

**2.1. Evaluation of DPPH Free Radical-scavenging.‎**

Radical scavenging activity of **5a, 5b, 12a**, and **12b** was measured by DPPH (2, 2-diphenyl-1-picrylhydrazyl) radical scavenging test according to the reported method [S3]. Different concentrations of **5a, 5b, 12a**, and **12b** (200–1000 ppm) were added to an equal volume of methanolic solution of DPPH (1 mmol/L). The mixtures well shaken and then placed in a dark room. After 30 min at room temperature, the absorbance recorded at 517 nm. In the control sample, **5a, 5b, 12a**, and **12b** replaced with 3 mL methanol. Butylated hydroxytoluene (BHT) and 2-tertbutylhydroquinone (TBHQ) used as standard controls. The percentage inhibition of the DPPH radical was calculated according to the formula of Yen and Duh [S4].

**2.2. Fe^3+^ reducing power.‎**

The ability of compounds **5a, 5b, 12a**, and **12b** to reduce iron (III) was evaluated by the method of Yildirim et al. [S5] samples (1 mL) were mixed with 2.5 mL of phosphate buffer (0.2 mol/L, pH 6.6) and 2.5 mL of potassium ferricyanide (K_3_Fe(CN)_6_; 10g/L) and showed for 30 min at 50 8C. Then, 2.5 mL of trichloroacetic acid (10% w/v) added to the solution and centrifuged for 10 min. Finally, 2.5 mL of supernatant combined with 2.5 mL of distilled water and 0.5 mL FeCl_3_ (1 g/L). The absorbance of samples measured at 700 nm. Higher absorbance means higher reducing power. Each measurement carried out in triplicate. The data were analyzed by running one way analysis of variance (ANOVA) using SPSS software version 18.0. A one way ANOVA employed to evaluate difference in the mean value of samples and control. All mean separations performed by Duncan multiple range test using the significance level of 95% (P < 0.05).

**References**

[S1] R. Saundane and K. N. Mathada, *Monatsh Chem*., 2015, **146**, 1751.

[S2] M. Bidchol, A. Wilfred, P. Abhijna and R. Harish, *Food Bioprocess Tech*., 2011, **4**, 1137.

[S3] K. Shimada, K. Fujikawa, K. Yahara and T. Nakamura, *J. Agric. Food Chem*., 1992, **40**, 945.

[S4] G. C. Yen and P. D. Duh, *J. Agric. Food Chem*., 1994, **42**, 629.

[S5] Yildirim, A. Mavi and A. A. Kara, *J. Agric. Food Chem*., 2001, **49**, 4083.


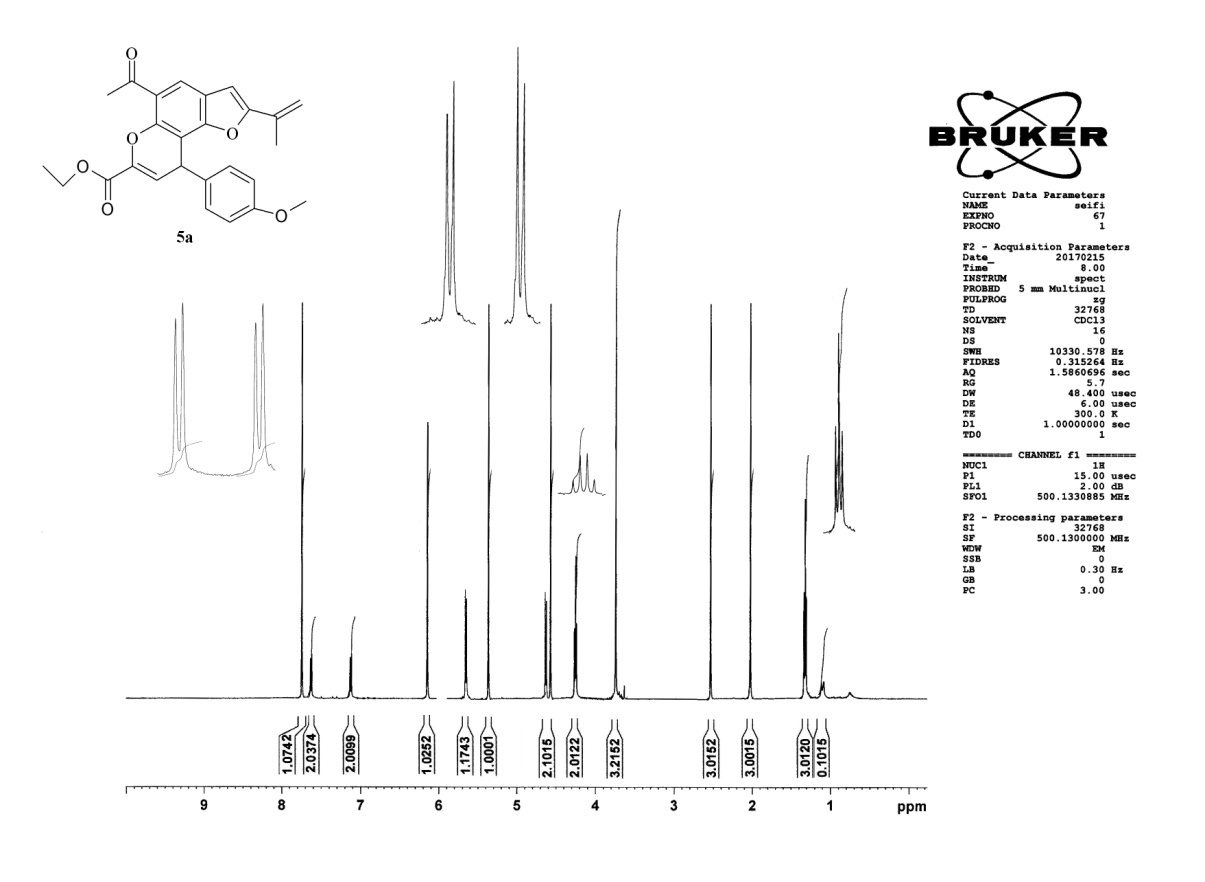


**Figure S3** (^1^H NMR spectrum of 5a)


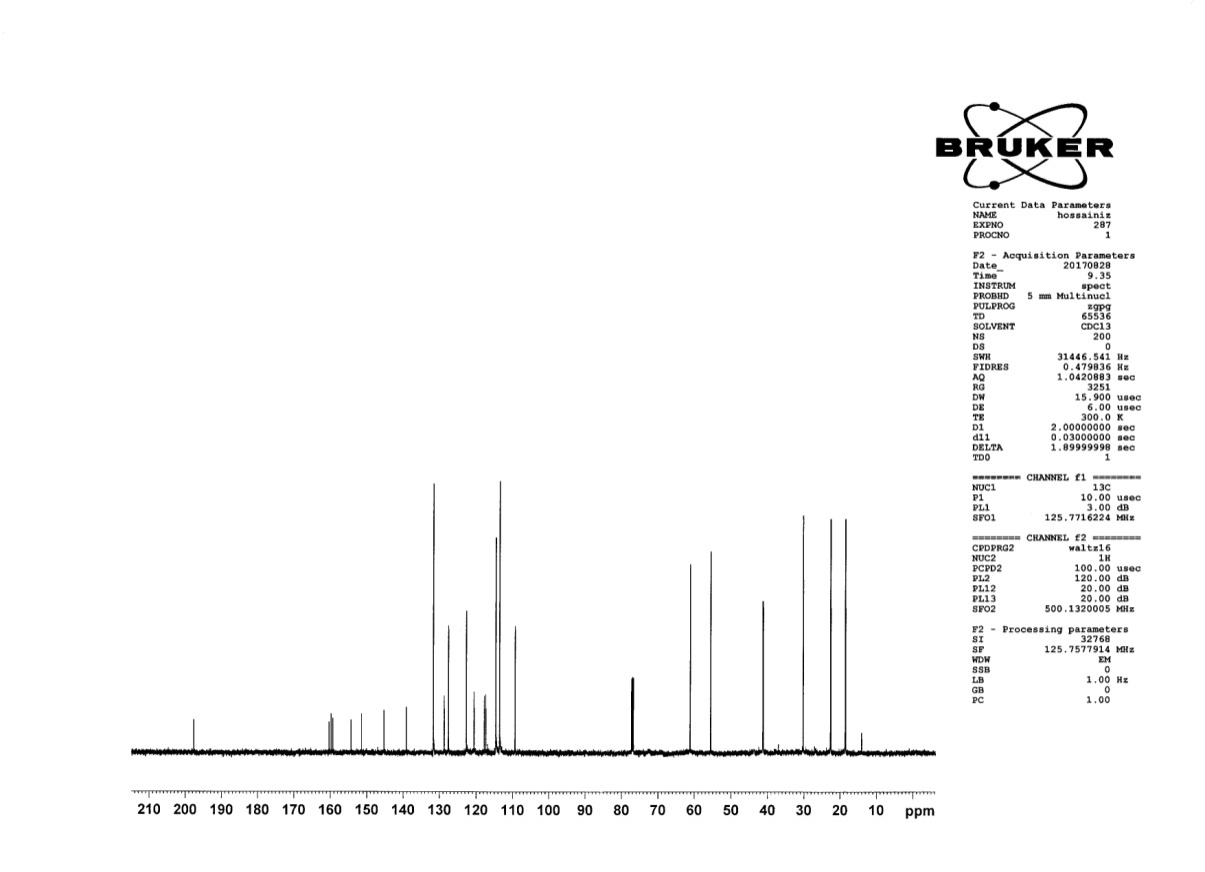


**Figure S4** (^13^C NMR spectrum of 5a)


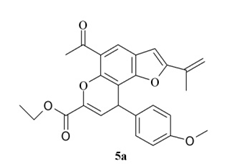

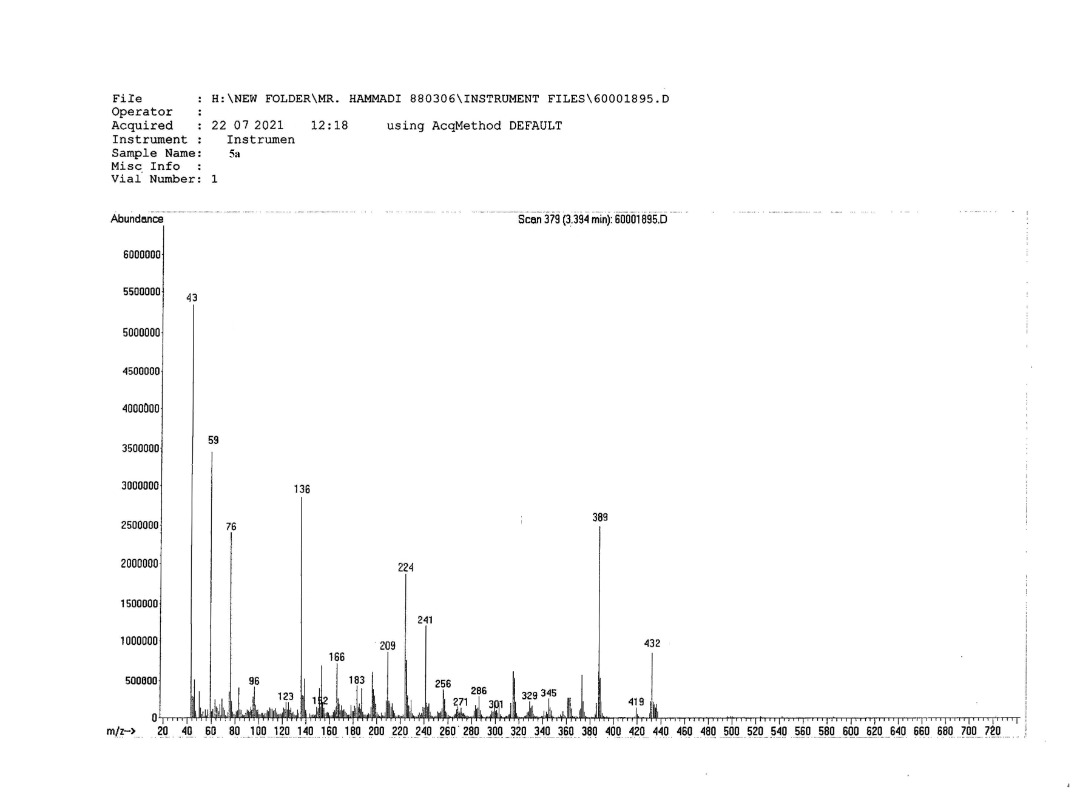


**Figure S5** (Mass spectrum of 5a)


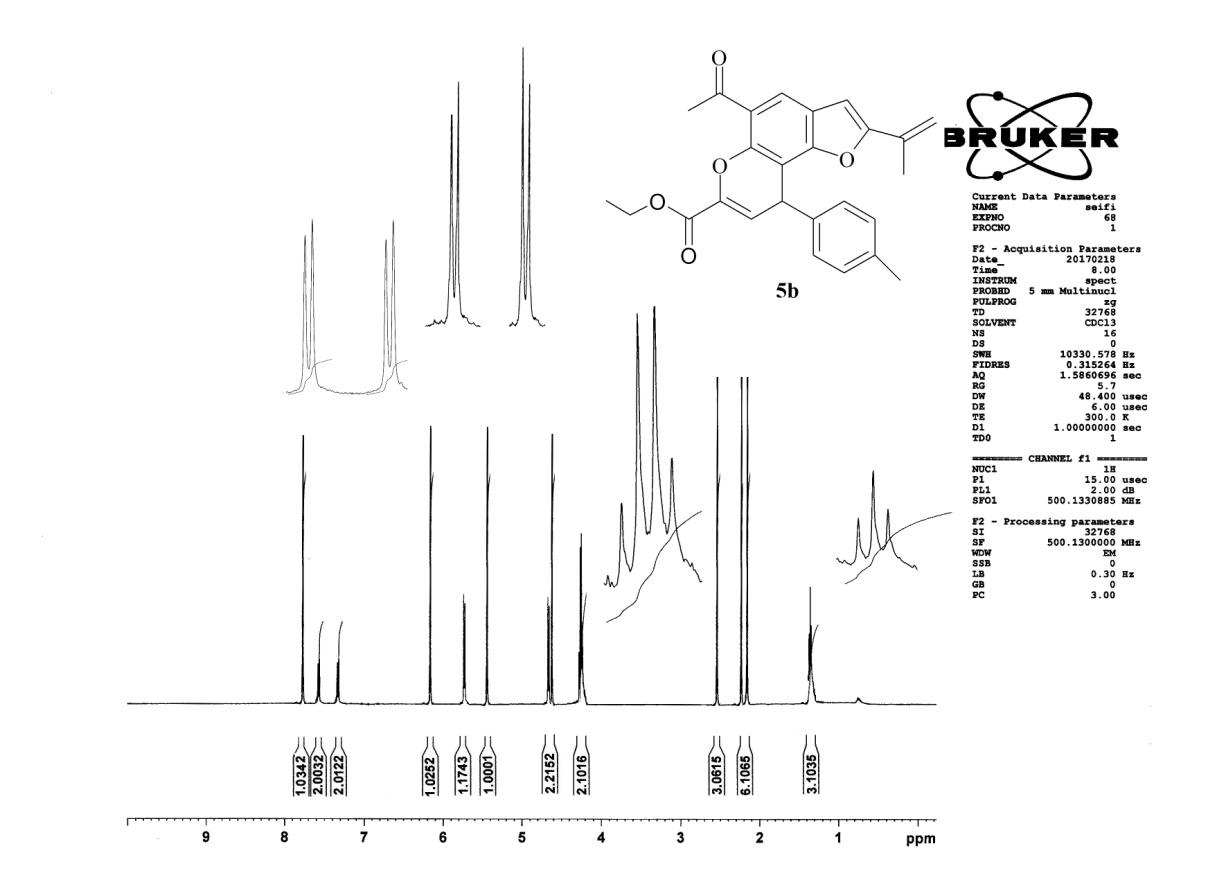


**Figure S6** (^1^H NMR spectrum of 5b)


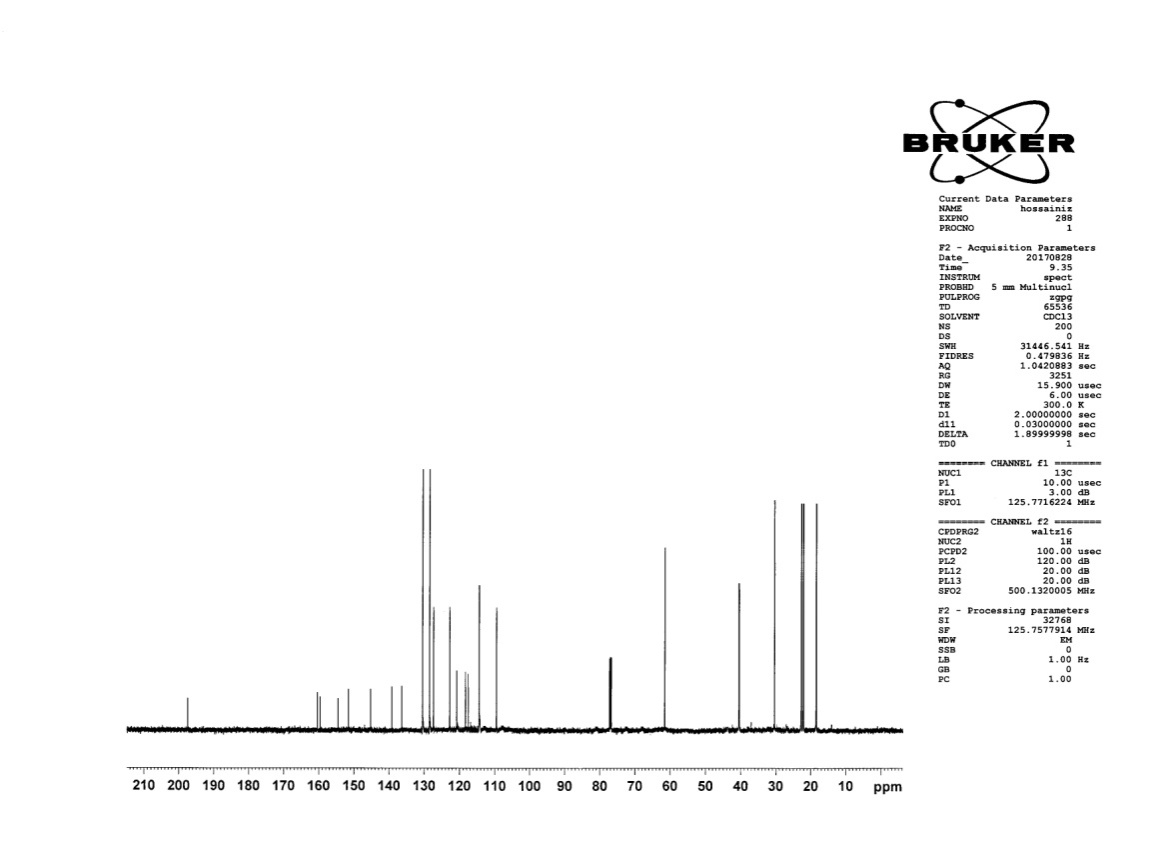


**Figure S7** (^13^C NMR spectrum of 5b)


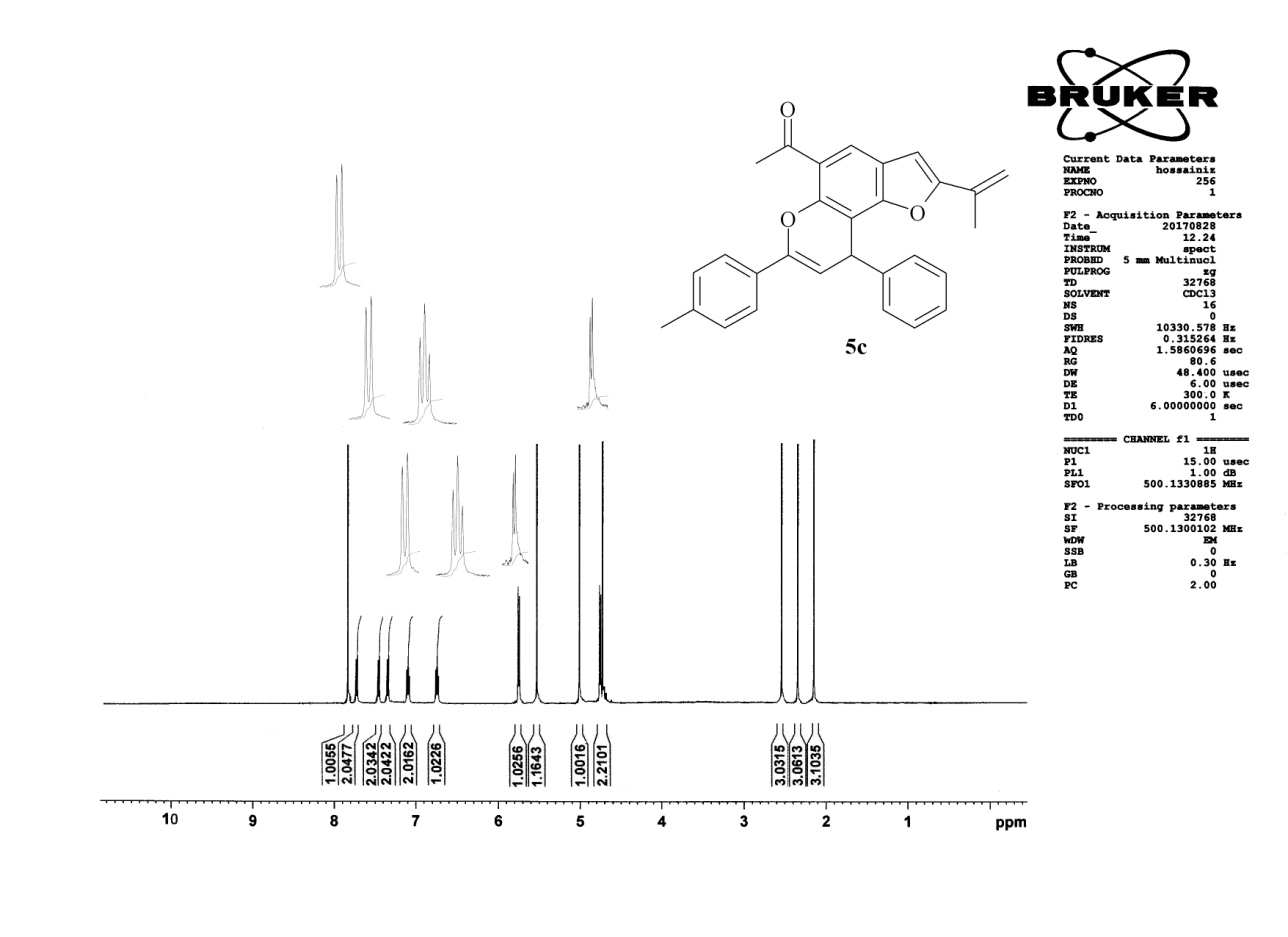


**Figure S8** (^1^H NMR spectrum of 5c)


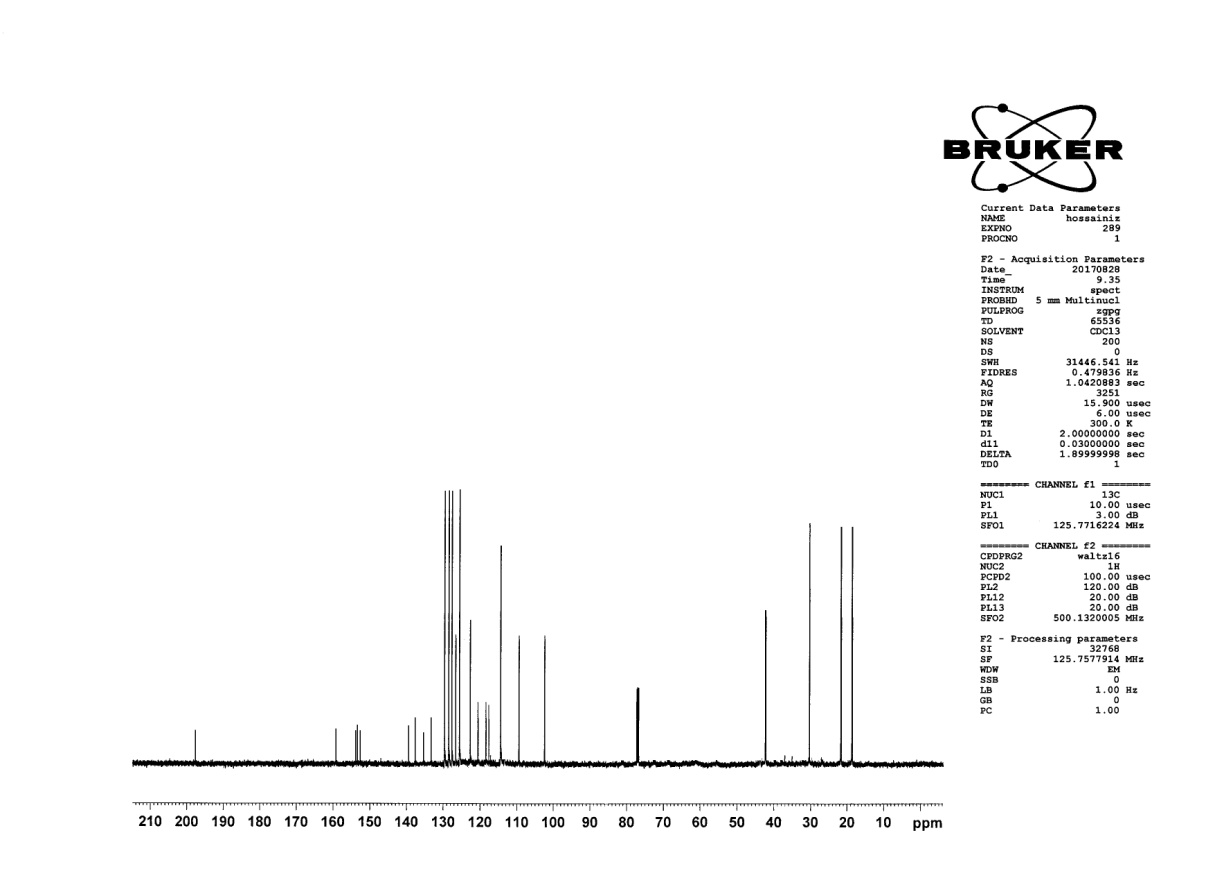


**Figure S9** (^13^C NMR spectrum of 5c)

**
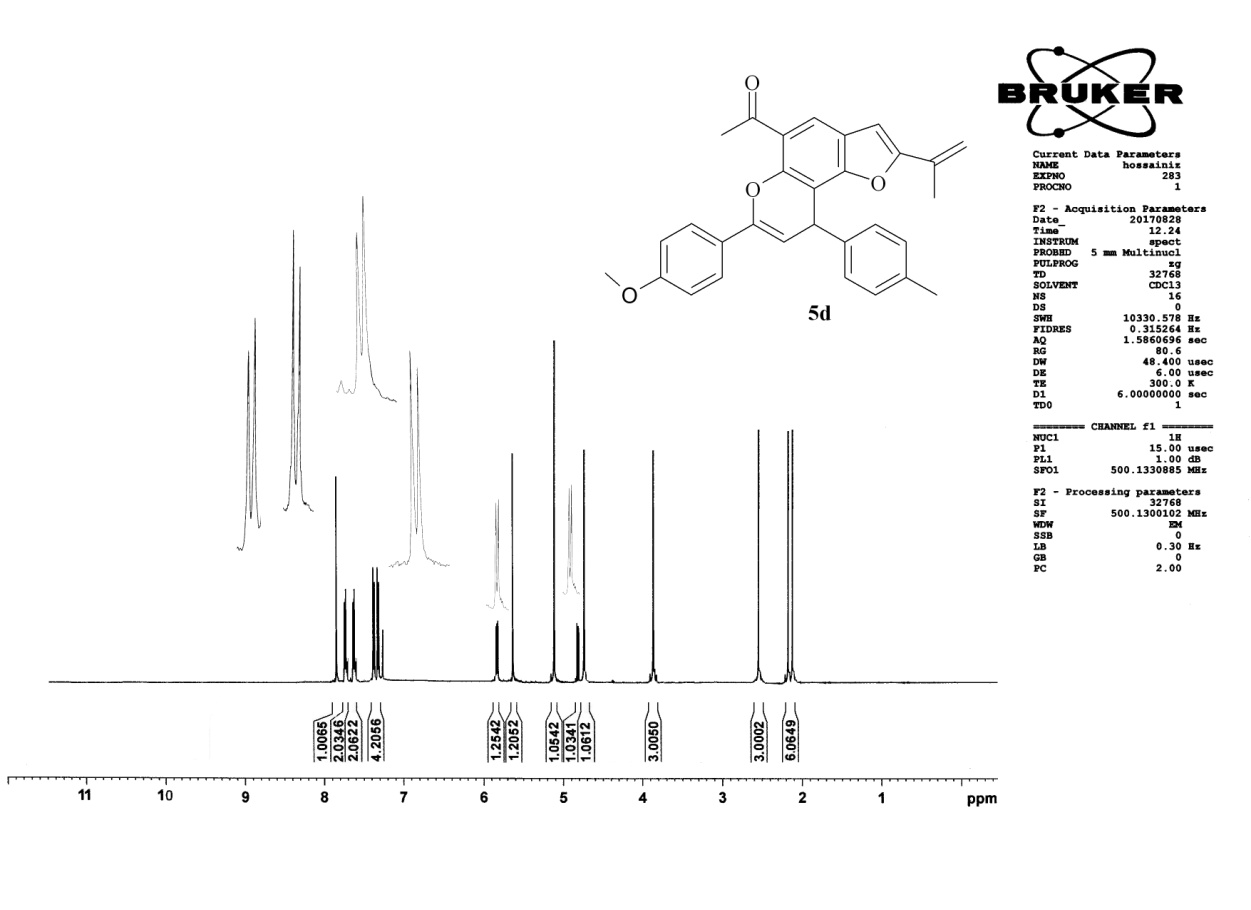
**

**Figure S10** (^1^H NMR spectrum of 5d)


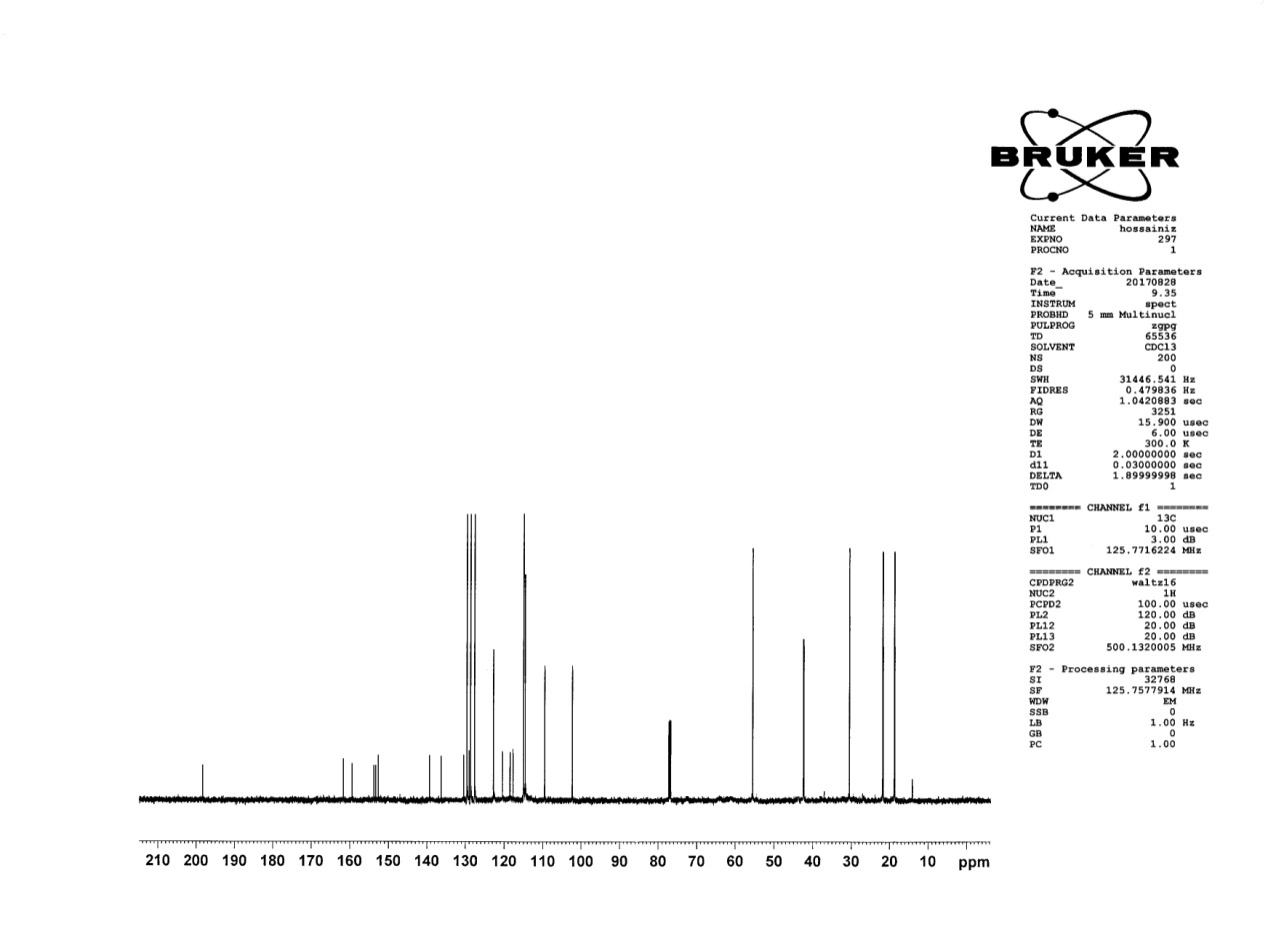


**Figure S11** (^13^C NMR spectrum of 5d)

**
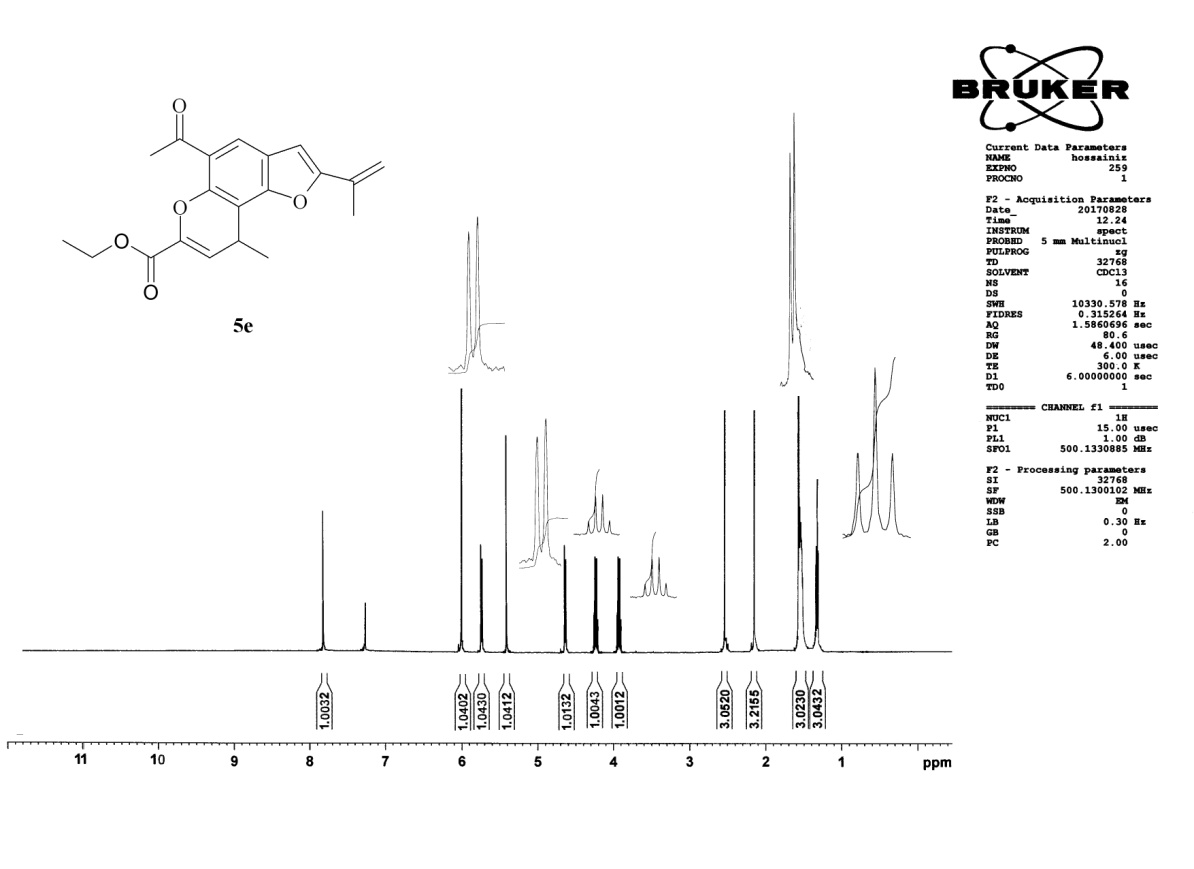
**

**Figure S12** (^1^H NMR spectrum of 5e)

**
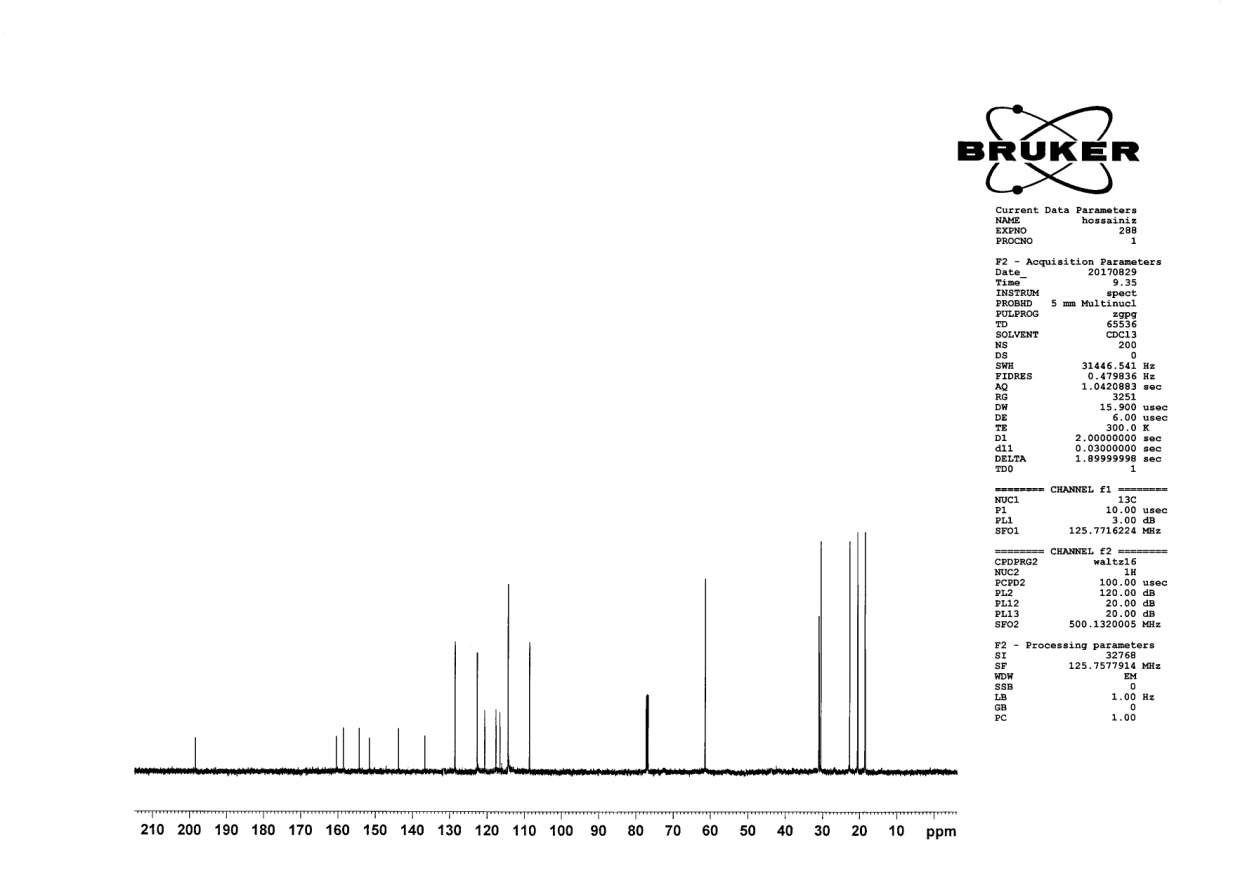
**

**Figure S13** (^13^C NMR spectrum of 5e)


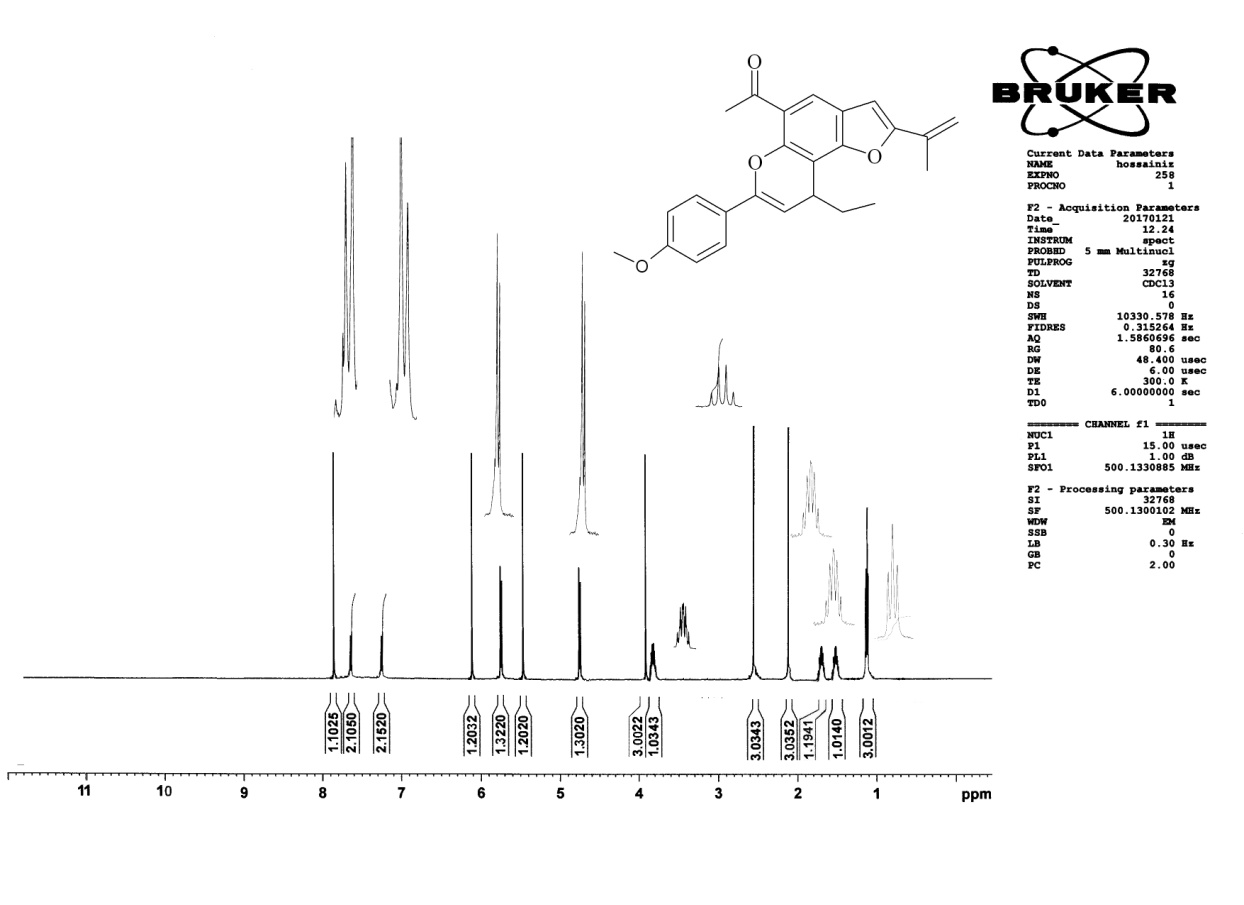


**Figure S14** (^1^H NMR spectrum of 5f)


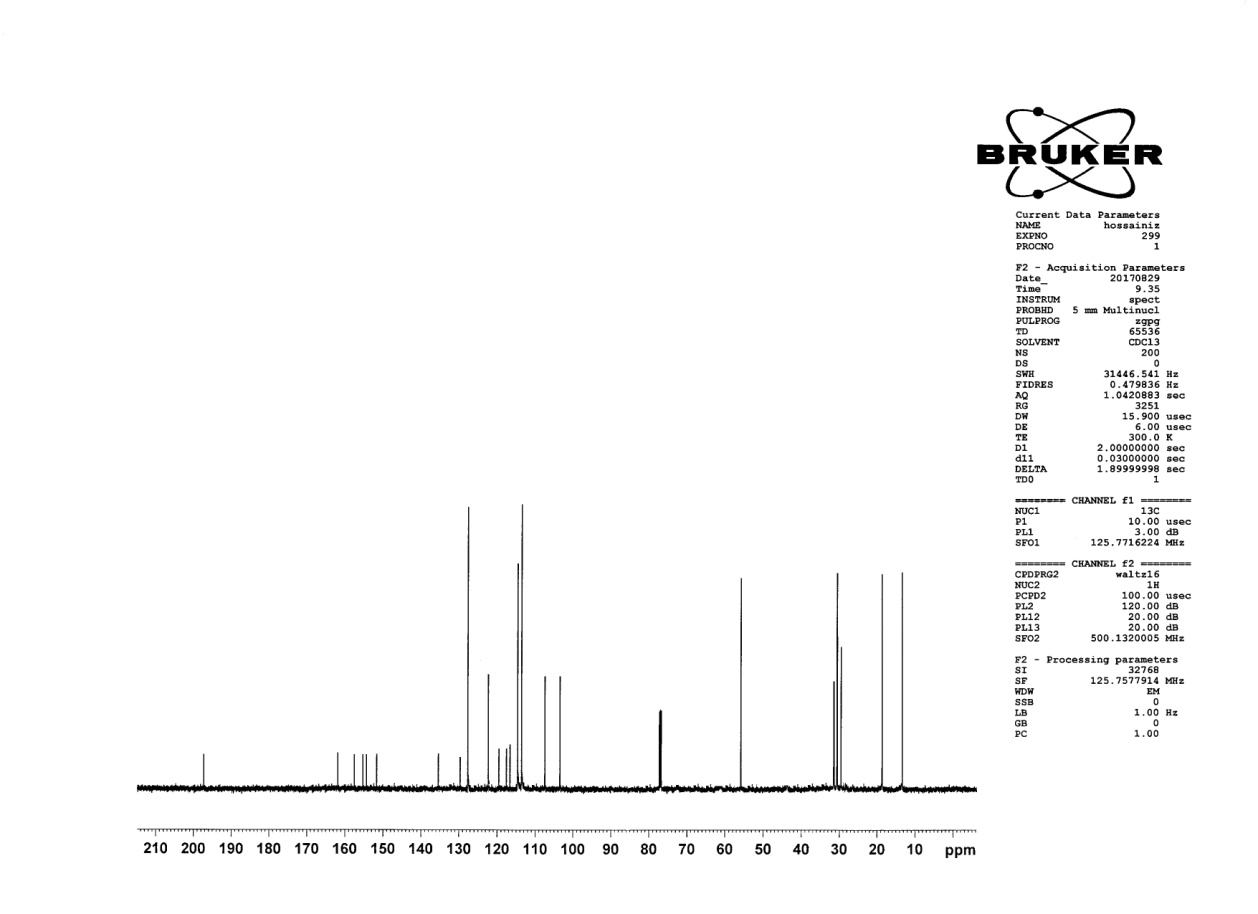


**Figure S15** (^13^C NMR spectrum of 5f)


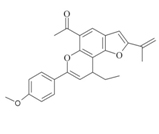

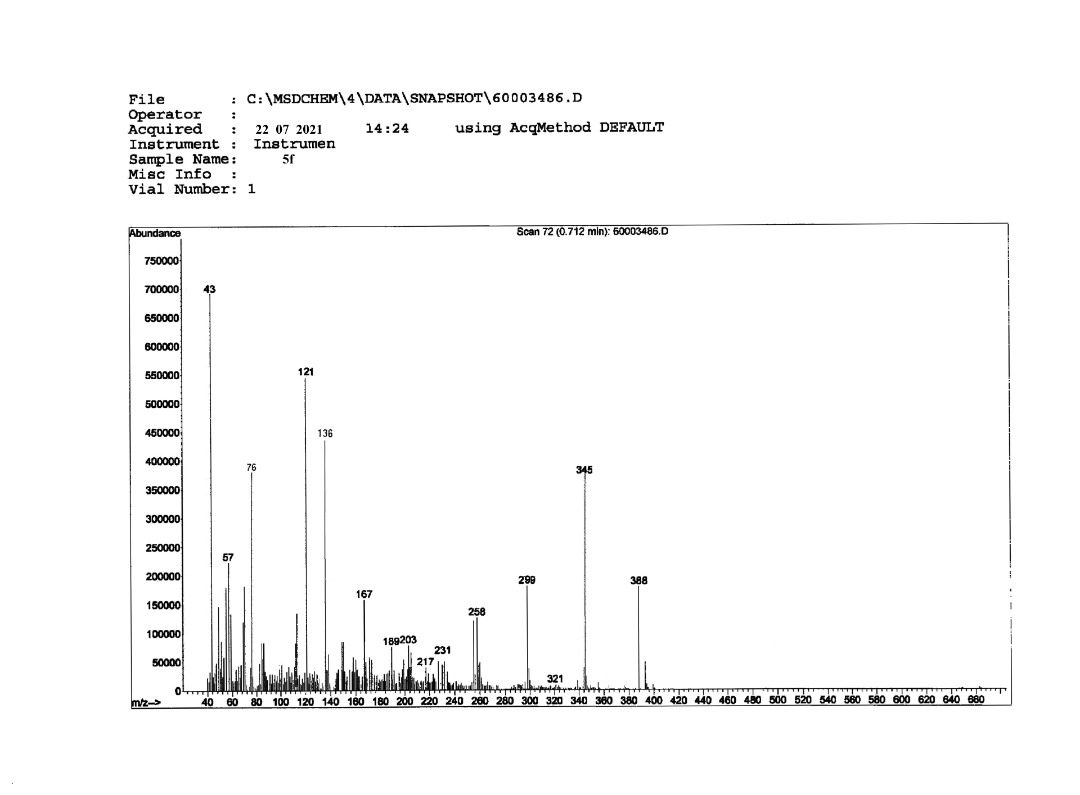


**Figure S16** (Mass spectrum of 5f)


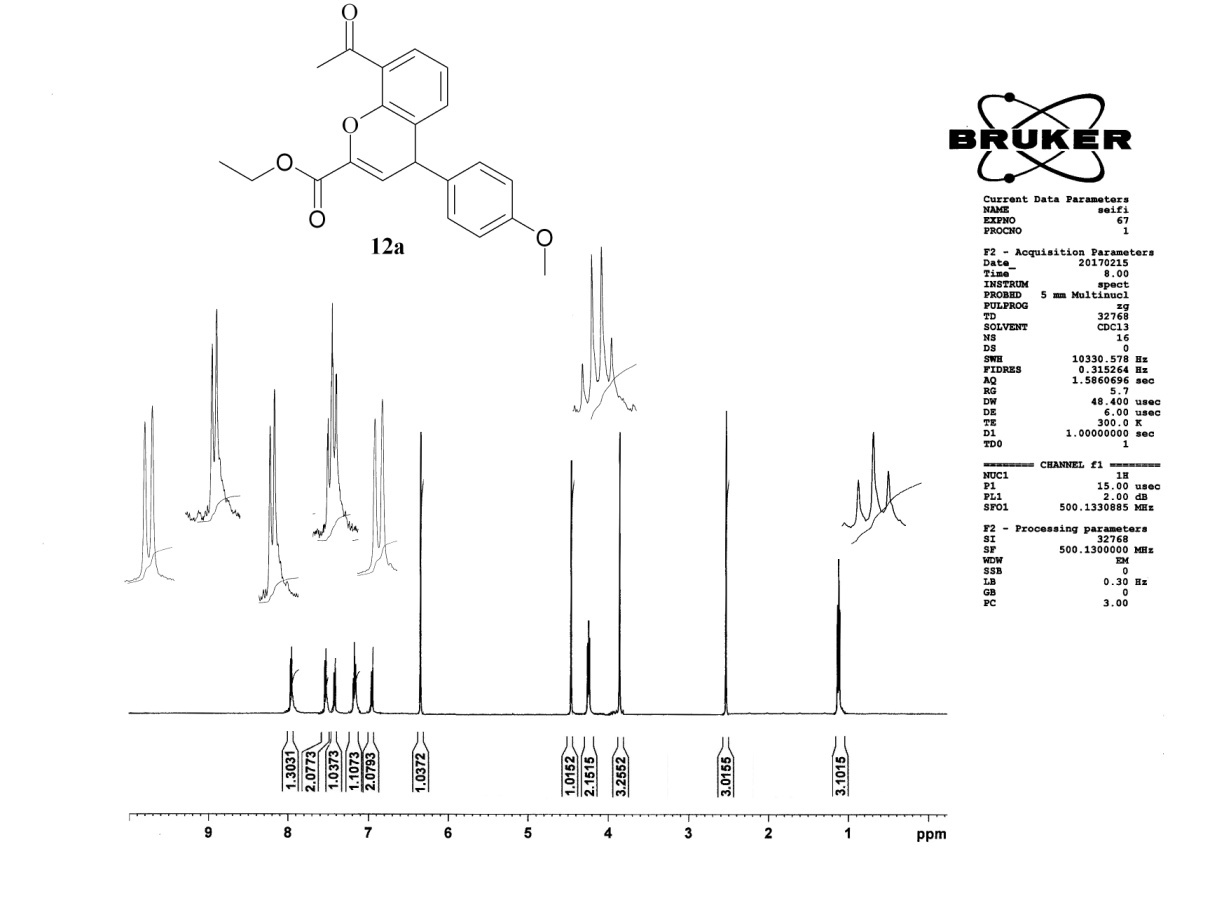


**Figure S17** (^1^H NMR spectrum of 12a)


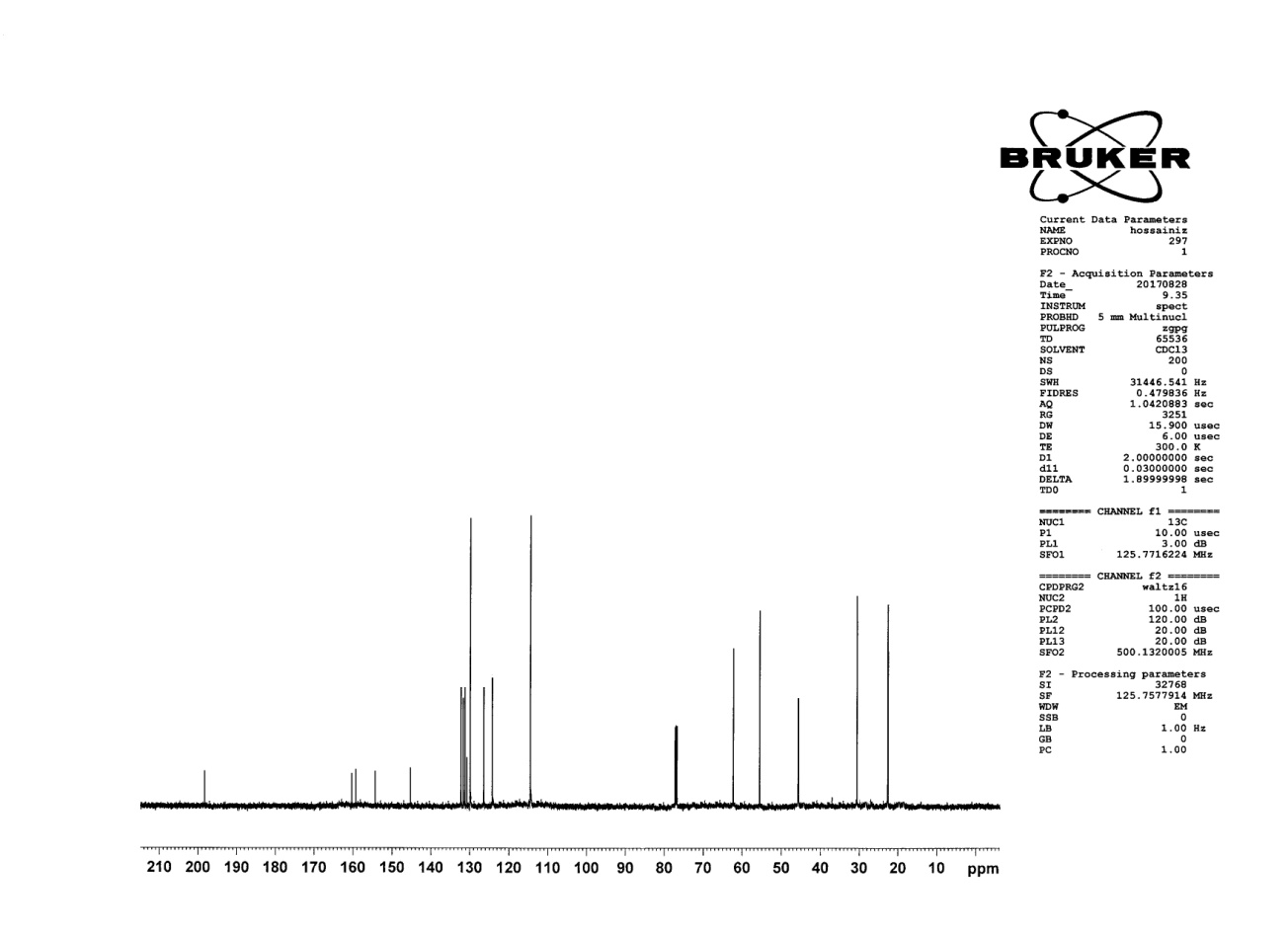


**Figure S18** (^13^C NMR spectrum of 12a)


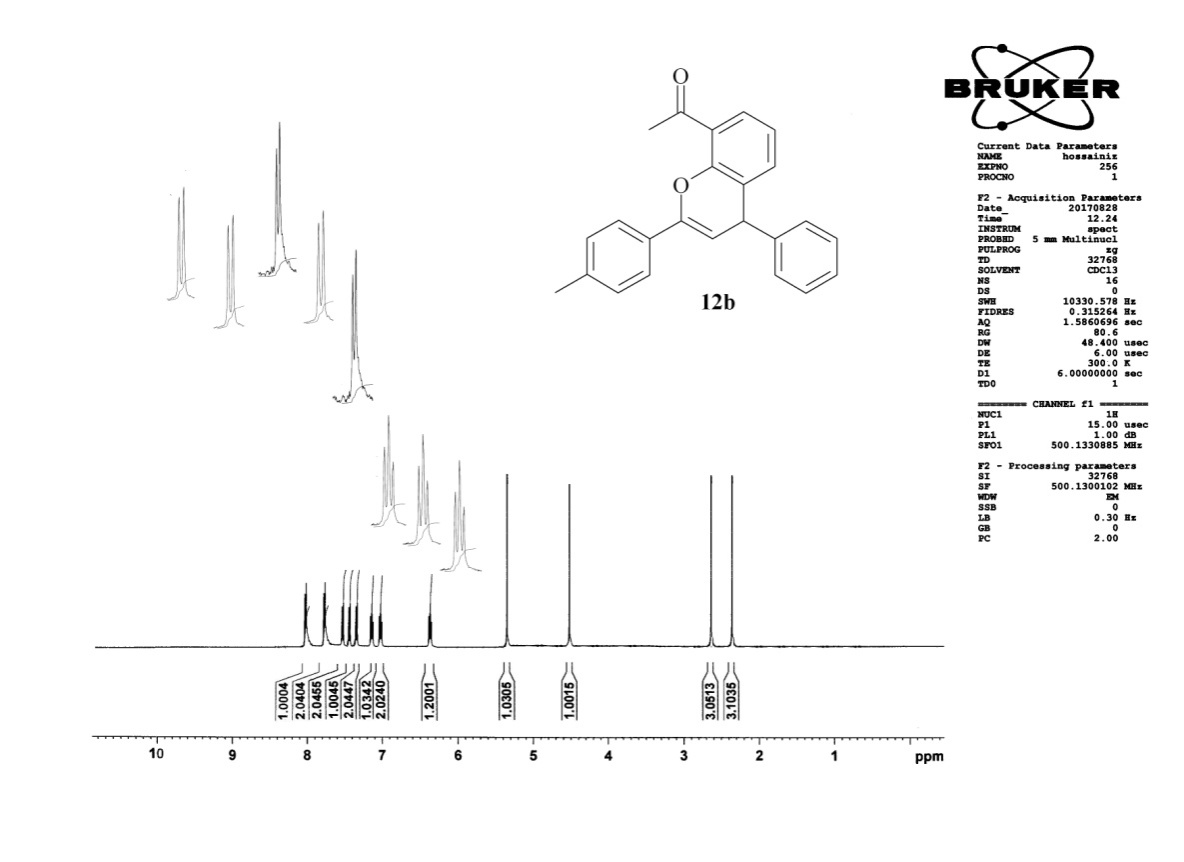


**Figure S19** (^1^H NMR spectrum of 12b)


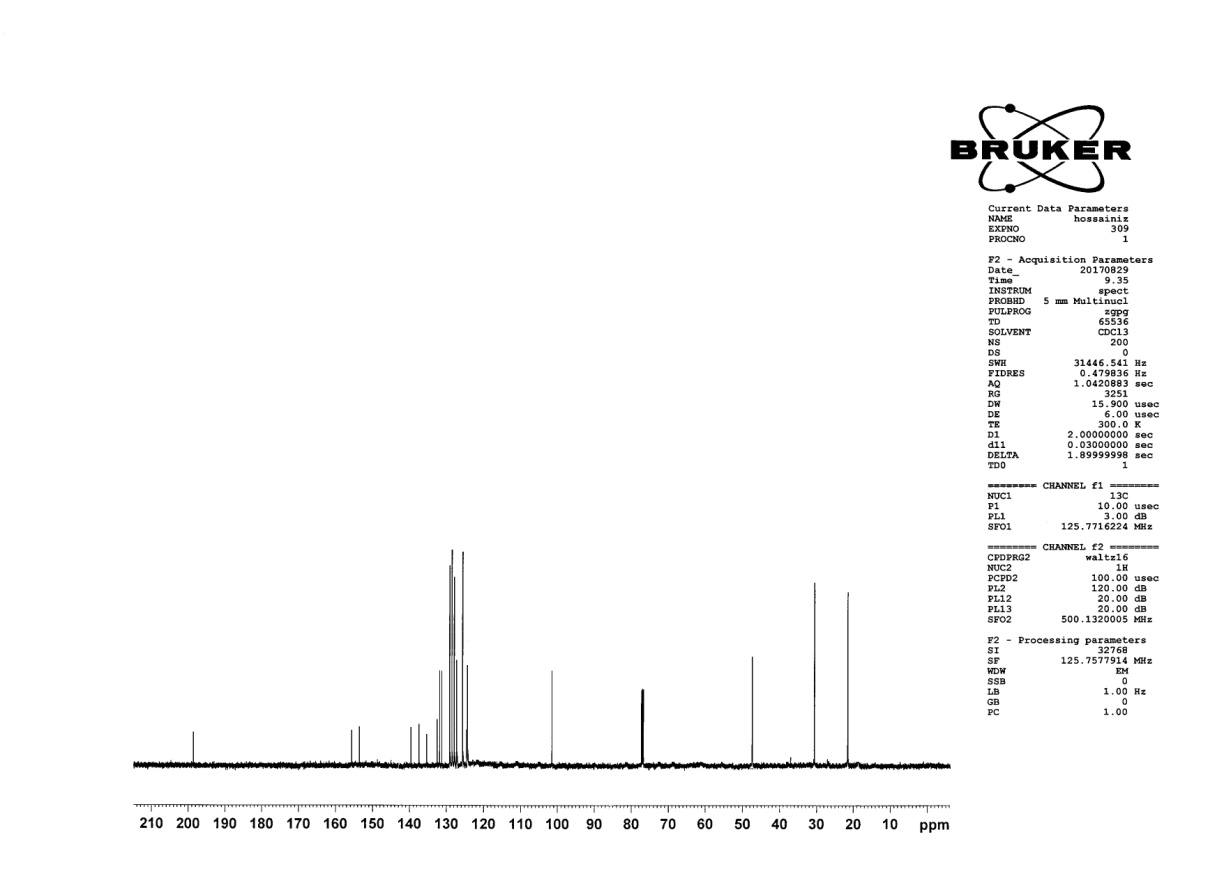


**Figure S20** (^13^C NMR spectrum of 12b)


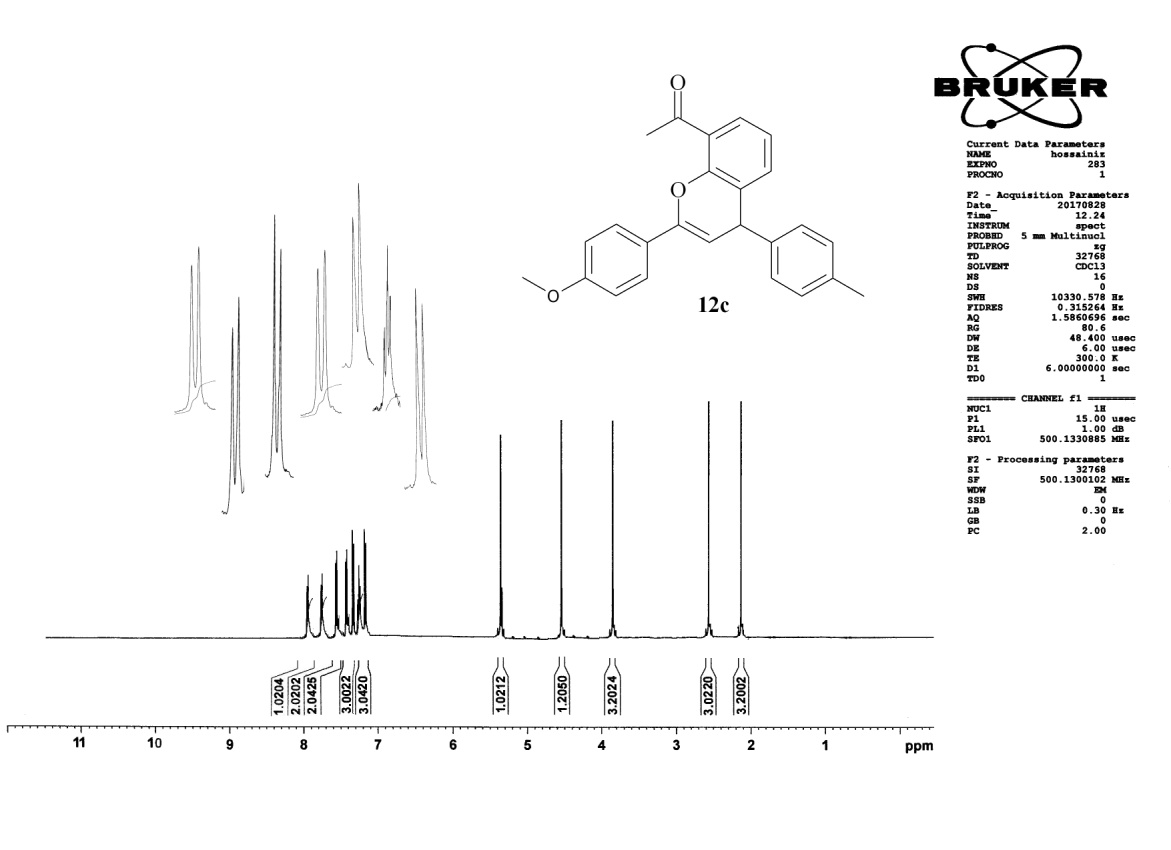


**Figure S21** (^1^H NMR spectrum of 12c)

**
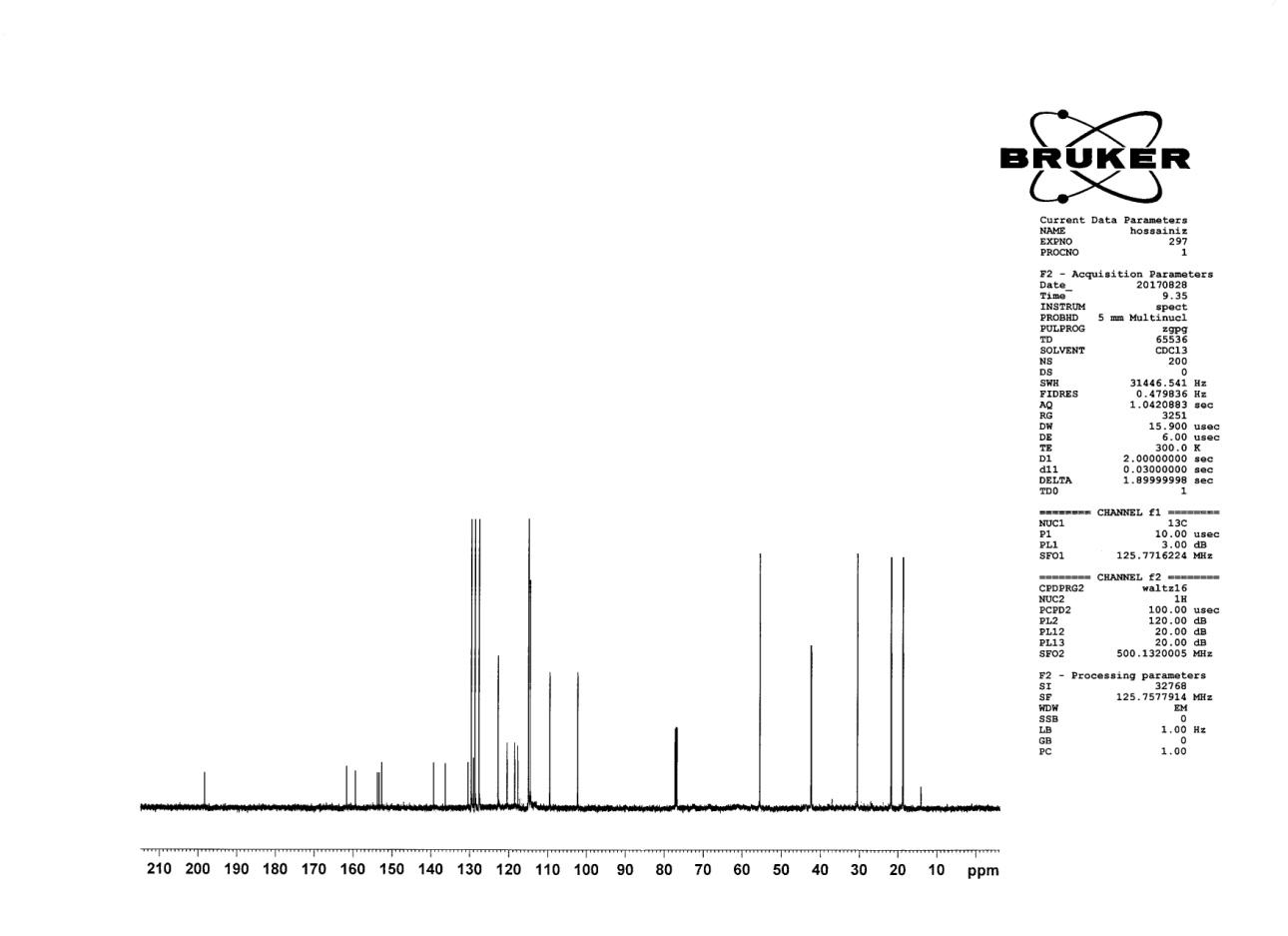
**

**Figure S22** (^13^C NMR spectrum of 12c)


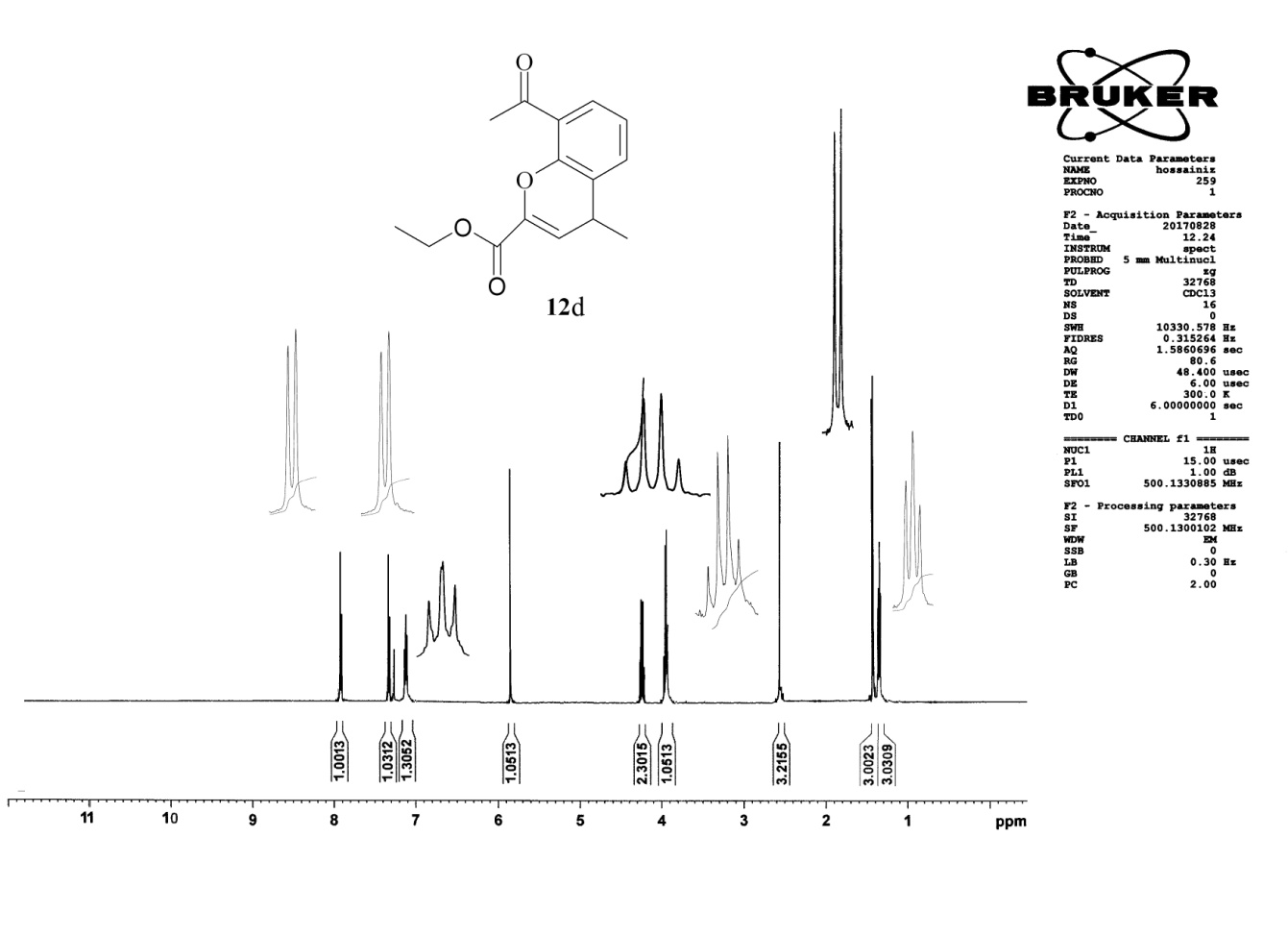


**Figure S23** (^1^H NMR spectrum of 12d)


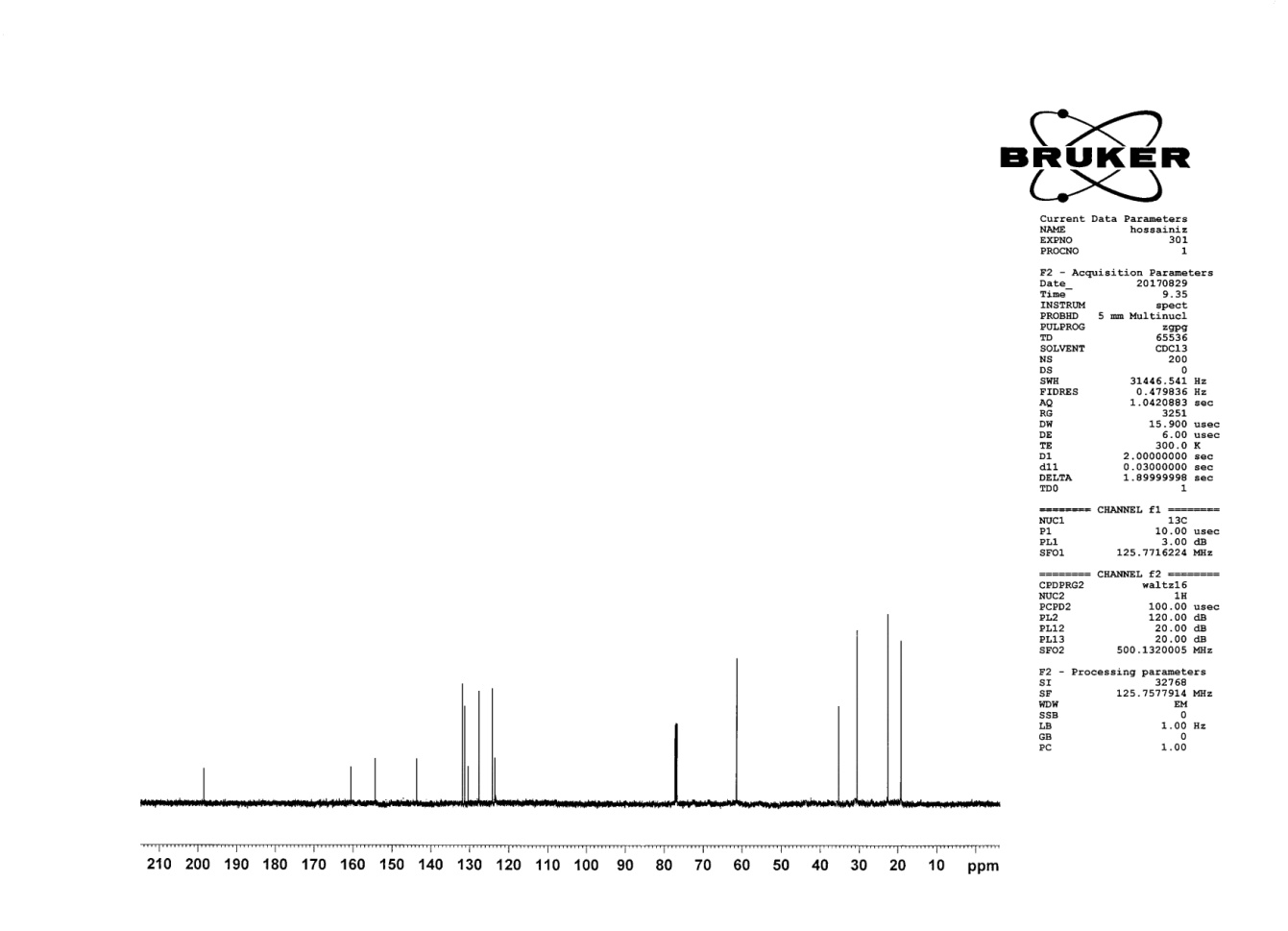


**Figure S24** (^13^C NMR spectrum of 12d)

**
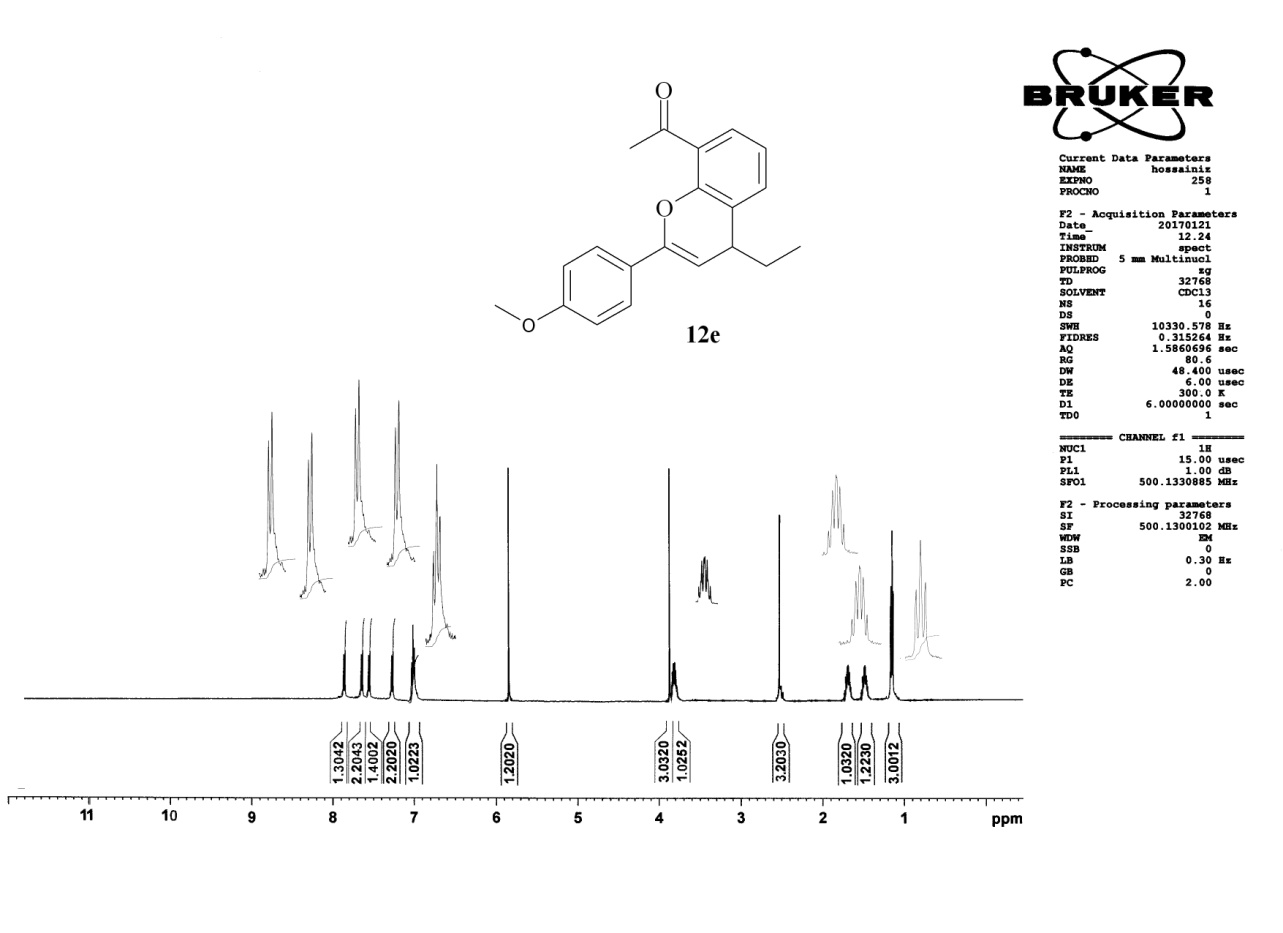
**

**Figure S25** (^1^H NMR spectrum of 12e)

**
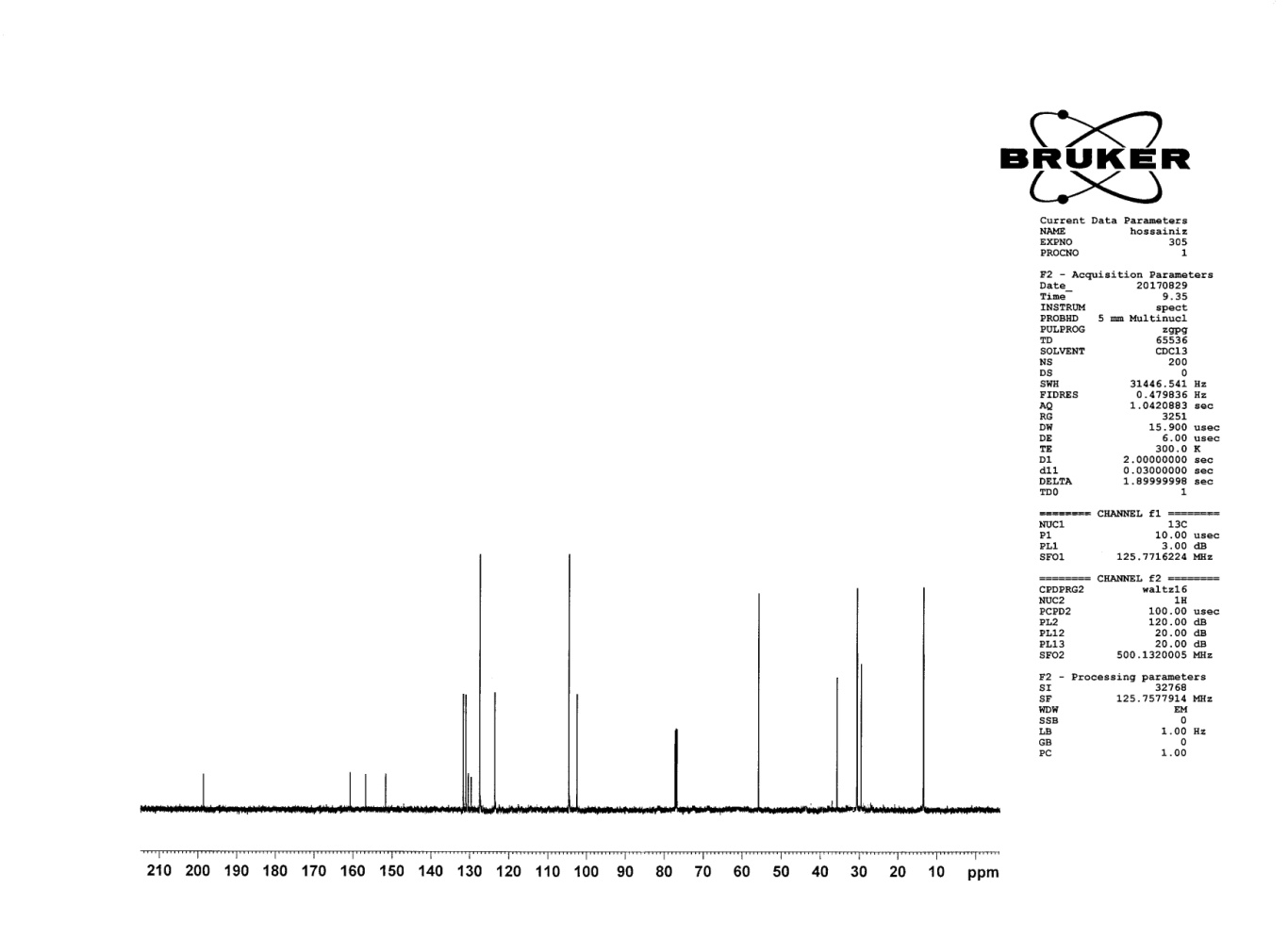
**

**Figure S26** (^13^C NMR spectrum of 12e)
